# Evaluation of semi-automated record screening methods for systematic reviews of prognosis studies and intervention studies

**DOI:** 10.1017/rsm.2025.10025

**Published:** 2025-07-22

**Authors:** Isa Spiero, Artuur M. Leeuwenberg, Karel G. M. Moons, Lotty Hooft, Johanna A. A. Damen

**Affiliations:** 1 Julius Center for Health Sciences and Primary Care, University Medical Center Utrecht, Utrecht University, Utrecht, the Netherlands; 2 Cochrane Netherlands, Julius Center for Health Sciences and Primary Care, University Medical Center Utrecht, Utrecht University, Utrecht, the Netherlands

**Keywords:** active learning, clinical guideline development, large language models, prioritized screening, semi-automation, systematic reviews

## Abstract

Systematic reviews (SRs) synthesize evidence through a rigorous, labor-intensive, and costly process. To accelerate the title–abstract screening phase of SRs, several artificial intelligence (AI)-based semi-automated screening tools have been developed to reduce workload by prioritizing relevant records. However, their performance is primarily evaluated for SRs of intervention studies, which generally have well-structured abstracts. Here, we evaluate whether screening tool performance is equally effective for SRs of prognosis studies that have larger heterogeneity between abstracts. We conducted retrospective simulations on prognosis and intervention reviews using a screening tool (ASReview). We also evaluated the effects of review scope (i.e., breadth of the research question), number of (relevant) records, and modeling methods within the tool. Performance was assessed in terms of recall (i.e., sensitivity), precision at 95% recall (i.e., positive predictive value at 95% recall), and workload reduction (work saved over sampling at 95% recall [WSS@95%]). The WSS@95% was slightly worse for prognosis reviews (range: 0.324–0.597) than for intervention reviews (range: 0.613–0.895). The precision was higher for prognosis (range: 0.115–0.400) compared to intervention reviews (range: 0.024–0.057). These differences were primarily due to the larger number of relevant records in the prognosis reviews. The modeling methods and the scope of the prognosis review did not significantly impact tool performance. We conclude that the larger abstract heterogeneity of prognosis studies does not substantially affect the effectiveness of screening tools for SRs of prognosis. Further evaluation studies including a standardized evaluation framework are needed to enable prospective decisions on the reliable use of screening tools.

## Highlights

### What is already known?

- The conduct of SRs can be accelerated by AI-based semi-automated screening tools in which active learning and machine learning are combined to prioritize potentially relevant abstracts.
- Previous studies using such tools have already shown substantial workload reductions in title–abstract screening. However, these tools are most often tested for reviews of intervention studies.

### What is new?

- We evaluated the performance of a screening tool for SRs of prognosis studies as the larger heterogeneity between abstracts of prognosis studies could negatively affect the tool performance.
- The performance of the tool was only slightly decreased for prognosis reviews compared to intervention reviews. The scope of the review research question and modeling methods within the tool only slightly affected the screening tool performance, while the effect of the number of (relevant) records in the dataset was strongly related to tool performance.

### Potential impact for RSM readers

- With this study, we demonstrate that screening tools are almost equally effective for prognosis reviews as compared to intervention reviews.
- We also gained insight into some additional characteristics of reviews and/or tool settings that affect tool performance. These insights in the context in which tools may perform well could eventually enable users to prospectively decide the suitability of tool use for a given review.

## 1 Introduction

Systematic reviews (SRs) and clinical guidelines form an essential part of evidence-based medicine by presenting thorough literature searches to collect and summarize all relevant primary studies to answer a given research question. It is essential to maximize the sensitivity of the literature search to reduce the amount of bias in the conclusions of the review or guideline. Hereto, a broad literature search in electronic databases is typically applied, frequently resulting in large numbers of records that need to be screened for relevance, first by title–abstract screening followed by full text screening. Conducted manually, this process is error prone, labor-intensive, and time-consuming, with the average review taking 1–2 years until completion.^1^ Moreover, as new evidence emerges faster by the increasing number of studies that are published each year,^2^ a SR or guideline may already be outdated by the time it is published.^3^

After one of the first attempts to accelerate the screening process with automated classification of titles and abstracts by Cohen *et al.* ^4^ many artificial intelligence (AI)-based (semi-)automated screening tools and algorithms have been developed.^5^ These screening tools combine feature extraction models that draw relevant features from the titles and abstracts with classification models that subsequently use these features to predict probabilities of relevance for each study. In practice, such tools are implemented either fully automated by providing the user with a binary outcome label (i.e., “relevant” or “irrelevant”) for each of the records or, most often, semi-automated by not dichotomizing but rather ranking or prioritizing the records based on the predicted probability of relevance.^6^ In the latter, records are ranked according to the predicted relevance, and researchers screen the records in that order. The screening tool is iteratively retrained by the decisions that the researcher makes, and the records are reranked accordingly. This is also called “active learning” and can be divided into certainty based (in which the researcher is first presented with the records that the algorithm is most certain about) and uncertainty sampling (in which the algorithm presents the records that it is least certain about).^7^ By using this procedure, semi-automated screening tools can have one of several functions such as (1) increasing rate of screening and/or improving workflow by prioritizing relevant records, (2) functioning as a second screener, and (3) reducing the number of records to screen by excluding records predicted to be irrelevant.^5^ Currently, most tools are semi-automatic and mainly function as a second screener or as improvement of workflow by prioritization during manual screening.^7^

Performance evaluations of semi-automated screening tools have shown an average workload reduction of about 50%, but this varies greatly across contexts.^6^^,^ ^8^ This heterogeneity in tool performance is presumably related to factors such as the tool and its modeling methods,^9^ number of records to be screened for the review,^6^^,^ ^10^ number of labeled records used for training the models within the tool,^8^^,^ ^11^ complexity/breadth of the research question,^12^^,^ ^13^ abstract structure and coherence,^14^ number of relevant records,^8^^,^ ^15^ review type (e.g., intervention, prognosis, or diagnosis), and/or medical topic .^15^^,^ ^16^ However, the validation of a semi-automated screening tool is often based on performance evaluations with only one or just a few SRs,^10^ and each evaluation study uses different methods as there is no standardized way for evaluation.^5^ Therefore, it is difficult to make valid comparisons across evaluation studies to assess the effect of aforementioned factors on tool performance and to subsequently decide prospectively whether tool use is suitable in a given setting.

Within the medical field, tools have mostly been validated on (therapeutic or preventive) intervention reviews, especially concerning randomized clinical trials (RCTs). For RCTs, there is typically better adherence to strict abstract reporting guidelines,^11^ as compared to, for example, prognosis and diagnosis studies. Therefore, poor generalizability of performance to these types of studies is expected.^17^ For diagnostic test accuracy reviews, for example, it was already suspected that the unreliable identification of eligible records by semi-automated screening was caused by the inconsistent terminology used in the abstracts of these studies.^12^ Similarly, reports of prognosis studies are even more variable, and this heterogeneity between abstracts can even further increase for prognosis reviews that have topics with broader scopes (i.e., a broader research question to be answered by the review). For such broad scopes, a broader search strategy is typically applied, resulting in a larger number and a larger variability in retrieved records. In such cases, the algorithms of the tools may have more difficulty to find the features needed to predict a probability of relevance. At the same time, prognosis reviews generally have more records to be screened based on the search strategy,^13^ and the potential workload reduction that can be obtained by using a semi-automated tool for these reviews is thereby higher.

We evaluated whether the performance of title–abstract screening methods is equally sufficient for SRs of prognosis studies compared to intervention studies. We illustrate this by using a currently available screening tool as a case study. Additionally, we investigated whether factors such as the scope of the review, the number of (relevant) records of the review, and the modeling methods used in the tool contribute to performance differences between these review types.

## 2 Methods

### 2.1 Title–abstract screening tool

**Figure 1:**
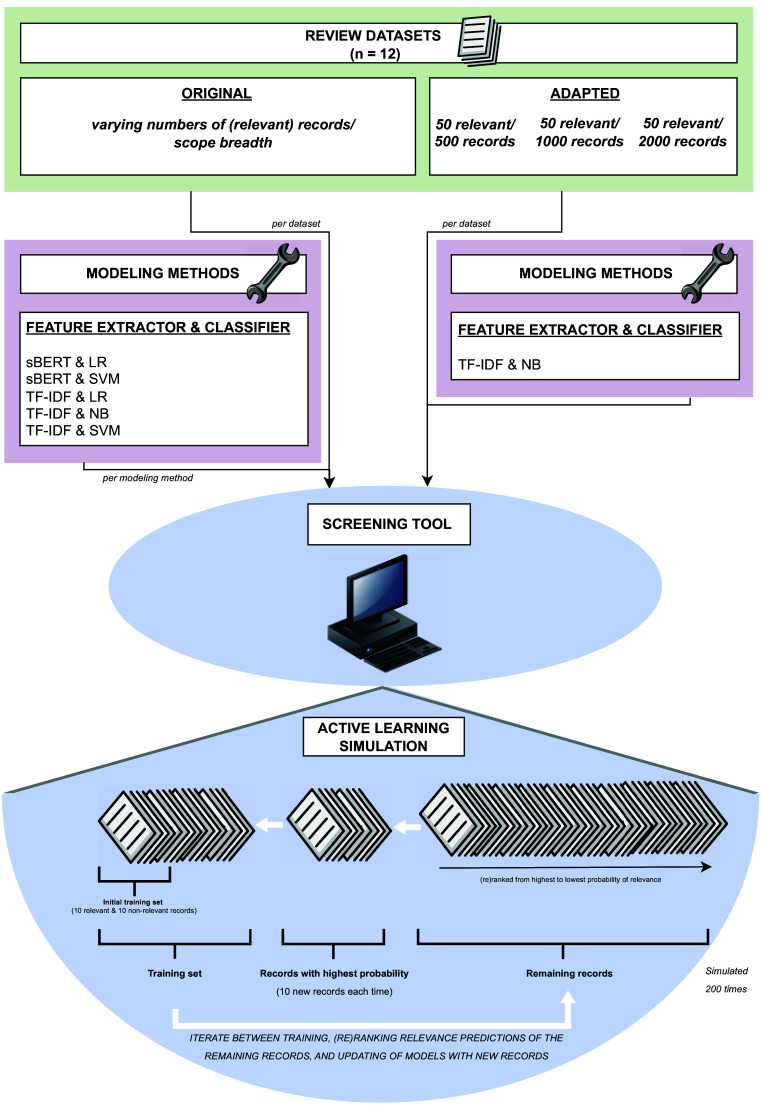
Overview of the active learning simulations that were conducted to evaluate the performance of a title–abstract screening tool. The scope breadth of the datasets, the modeling methods, and the number of (relevant) records were varied.

From the currently available AI-based semi-automated screening tools for title–abstract screening, the ASReview tool^15^ (hereafter called the screening tool) was chosen to compare the performance of semi-automated ranking algorithms when applied to SRs of prognosis and intervention studies. As a free, open-source tool based on Python, it allows researchers to flexibly conduct large amounts of simulations on screening tool use in contrast to other tools that are not open source. The tool has been evaluated for previous reviews including reviews within the medical domain.^16^ Several options are offered by the tool to perform simulation studies on labeled review datasets, with different feature extraction, classification, and query models among others to be chosen by the user. The classification models include methods such as support vector machines (SVMs) and naive Bayes (NB) that are also incorporated in other commonly used screening tools (such as AbstrackR,^18^ DistillerAI,^19^ EPPI-Reviewer,^20^ and Rayyan^21^) and could thereby give an indication of the performance of the algorithms within these tools as well. By using a labeled dataset and the chosen modeling methods, the screening tool conducts active learning in which the models are iteratively retrained to predict probabilities of relevance based on the titles and abstracts of the records and uses these probabilities to rank and prioritize the records to the user accordingly (Figure 1).

**Table 1:** Description of the review datasets used for evaluating the performance of a semi-automated title–abstract screening tool

Review	Total records	Relevant records (title–abstract screening) (%)	Final included records (full text screening)	Reference	Title
Prognosis reviews					
1^a^	2,482	312 (12.6)	152	Andaur Navarro *et al.* ^22^	Completeness of reporting of clinical prediction models developed using supervised machine learning: a systematic review
2^c^	777	91 (11.7)	38	Damen *et al.* ^23^	Performance of the Framingham risk models and pooled cohort equations for predicting 10-year risk of cardiovascular disease: a systematic review and meta-analysis
3^a^	4,871	347 (7.1)	146	Heus *et al.* ^24^	Poor reporting of multivariable prediction model studies: towards a targeted implementation strategy of the TRIPOD statement
4^b^	4,274	377 (8.8)	88	Pladet *et al.* ^25^	Prognostic models for mortality risk in patients requiring ECMO
5^b^	10,664	953 (8.9)	114	Takada *et al.* ^26^	Prognostic models for radiation-induced complications after radiotherapy in head and neck cancer patients
6^c^	3,999	1,064 (26.6)	107	Vernooij *et al.* ^27^	The comparative and added prognostic value of biomarkers to the Revised Cardiac Risk Index for preoperative prediction of major adverse cardiac events and all-cause mortality in patients who undergo noncardiac surgery
Intervention reviews					
1	9,160	54 (0.6)	25	Arikpo *et al.* ^28^ *	Educational interventions for improving primary caregiver complementary feeding practices for children aged 24 months and under
2	12,319	88 (0.7)	81	Chen *et al.* ^29^ *	First-line drugs inhibiting the renin angiotensin system versus other first-line antihypertensive drug classes for hypertension
3	2,815	37 (1.3)	10	Ijaz *et al.* ^30^ *	Psychological therapies for treatment-resistant depression in adults
4	3,874	52 (1.3)	12	Kahale *et al.* ^31^ *	Anticoagulation for people with cancer and central venous catheters
5	8,867	23 (0.3)	9	Kaufman *et al*.^32^ *	Face-to-face interventions for informing or educating parents about early childhood vaccination
6	5,392	112 (2.1)	68	Solomon *et al.* ^33^ *	Anti-vascular endothelial growth factor for neovascular age-related macular degeneration
A1 (Supplementary Material)	3,574	11 (0.3)	8	Kahn *et al.* ^34^ *	Interventions for implementation of thromboprophylaxis in hospitalized patients at risk for venous thromboembolism

* Retrieved from https://github.com/CLEF-TAR/tar/tree/master/2019-TAR/Task2.

a–c From broad to moderate to narrow review scope (i.e., the breadth of the research question of the review).

### 2.2 Datasets

To evaluate the performance of the screening tool, retrospective simulations were performed using a convenience sample of manually screened datasets of previously published prognosis (*n* = 6) and intervention reviews (*n* = 6) (Table 1). The reviews consist of a range of different topics and the number of records to be screened. The intervention review datasets are publicly available as being part of the Conference and Labs of the Evaluation Forum 2019 (CLEF 2019) challenge,^35^ and for the current study, a selection was made of the intervention reviews with the largest number of records (ranging from 2,815 to 12,319 records) to match (and reduce the potential effect of) the larger size of prognosis reviews (ranging from 777 to 10,664 records). Since the intervention review by Kahn *et al.* (2018) did not have enough relevant records to run simulations with default settings (as described under “Simulations”), the results for this review were not included in the main analysis but can be found in the Supplementary Material instead (Part IV: Figure A3 and Table A8 in the Supplementary Material).

Each of the datasets consists of records with manually labeled relevant/irrelevant labels based on title and abstract screening, which were used as the reference standard. The percentages of the manually included relevant records were generally low for the six intervention reviews (ranging from 0.3 to 2.1% of all screened records), while for prognosis reviews, the percentages were much higher (ranging from 7.1 to 26.6%). In addition to the division into review type (prognosis vs intervention), a further subdivision based on the scope of the review (i.e., breadth of the research question) was made for prognosis reviews to further examine the effect of abstract heterogeneity on screening tool performance. The scopes of the prognosis reviews could be roughly divided into: (A) broad scope reviews that had no restrictions for the population, outcome, or prediction model (prognosis reviews 1 and 3), (B) moderate scope reviews that had restrictions for population and outcome but not for prediction model (prognosis reviews 4 and 5), and (C) narrow scope reviews that had restrictions for the population, outcome, and prediction model (prognosis reviews 2 and 6).

### 2.3 Simulations

The title–abstract screening process was simulated with the datasets of these 12 reviews. To initiate a simulation, a predefined number of 10 relevant and 10 irrelevant records was randomly sampled from the review dataset by the tool. This sample functioned as the training dataset for the model to initiate the record ranking based on the probability of inclusion. For the ranking, the trained model was applied by the tool to the remaining records, and for each record, the probability of relevance was computed. The records were then ranked according to their relevance probability from highest to lowest (using the default query method of “maximum probability”) and simulated as being presented to the tool user, by starting with the record with the highest probability and using the relevant/irrelevant label of the respective record as the supposed decision by the user. After having presented the predefined number of 10 highest-ranking records, the model was updated while taking these 10 records and their labels into account. Then, the process of presenting the highest-ranking records, retrieving labels, and model updating was iterated until all records had passed. Since the initial training set (of 10 relevant and 10 irrelevant records) was sampled randomly, the entire process was repeated 200 times, in which each time, a new set of relevant and irrelevant records was sampled. Furthermore, since the number of relevant records differed per dataset, some datasets may have suffered from class imbalance (i.e., few relevant records compared to irrelevant records) more than others. In the screening tool, we set the balancing strategy that deals with this class imbalance to the tool’s default strategy (double balance).

Several variations within the simulations were introduced to test their effects. First, we used a sample of prognosis reviews consisting of different scope breadths, as described under *Datasets*. Broader scopes result in a larger variation in retrieved studies due to the broader search strategy, and therefore, more heterogeneity between titles and abstracts was expected. This can, in turn, influence the performance of the screening tool in distinguishing between relevant and irrelevant records.

Second, we varied the modeling methods within the tool. These modeling methods consist of algorithms which combine a feature extraction model with a classification model. A feature extraction model extracts features from the text, such as how often words occur in the abstracts. These features can then be used by a classification model that learns, based on these features, which abstracts are relevant and irrelevant and can then apply this knowledge to make predictions for unseen abstracts (e.g., it may learn that the occurrence of “high blood pressure” may be indicative that the abstract is likely relevant in a review on antihypertensive medication). For each of the datasets, we tested two different feature extraction models (term frequency-inverse document frequency [TF-IDF] and sentence Bidirectional Encoder Representations from Transformers [sBERT]) and three different classification models (NB, logistic regression, and SVM), leading to five different feature extraction and classification model combinations (as sBERT is not compatible with NB):

1. TF-IDF with NB (screening tool default),
2. TF-IDF with SVM,
3. TF-IDF with logistic regression,
4. sBERT with SVM,
5. sBERT with logistic regression.

Third, we varied the number of records in the review datasets to compare intervention reviews to prognosis reviews while ruling out the influence of review size and/or number of relevant records. The datasets were manually adapted to consist of a random sample of 500, 1,000, and 2,000 records, of which 50 were relevant. Each random sample from the original datasets was drawn five times. This was conducted only for the eight review datasets that allowed this in terms of available relevant records (i.e., intervention reviews 1, 2, 4, and 6 and prognosis reviews 3, 4, 5, and 6). Thus, an additional number of 24 manually adapted review datasets were simulated 200 times each, but here only with the tool’s default modeling settings (TF-IDF with NB).

### 2.4 Performance evaluation

A 2x2 cross-tabulation was used in which the reference standard of manual relevant/irrelevant labels was compared to predictions of the screening tool at a given percentage of records screened. The following sections describe the choice of metrics to quantify and compare the performance of the screening tool. The formulas for calculations and corresponding definitions of each metric can be found in the Supplementary Material (Part I).

The *recall (or sensitivity)* at a given number of records screened indicates the proportion of correctly classified positives by the tool. For SRs in the medical field, a recall of at least 95% is desired to prevent the introduction of bias in the results. However, at each given number of records screened, the number of false negatives is by default more likely to be lower when less relevant records are present in the dataset, and therefore, the recall is subsequently also more likely to be higher in such datasets compared to datasets that contain more relevant records. Thus, given this dependence of the recall on the number of relevant records that are present in the dataset, we additionally provided the *maximum achievable recall* at increasing numbers of records screened. This maximum value is dependent on the number of relevant records in the dataset; for example, if you have a tool with perfect performance (only returns true relevant records) and one screens 100 out of 1,000 records (of which 150 are relevant), the maximum achievable recall would be 100 (TP)/(100 (TP) + 50 (FN)) = 0.67, while if one screens 100 out of 1,000 records (of which 100 are relevant) the maximum achievable recall would be 100 (TP)/(100 (TP) + 0 (FN) = 1 already.

The *precision (or positive predictive value)* is defined by the proportion of correctly classified positives among the total number of classified positives and was calculated at the point that 95% recall was reached (precision@95%). The precision relates to the screening burden, and a higher value indicates that fewer irrelevant records need to be screened.

The *Work Saved over Sampling (WSS)* introduced by Cohen *et al.* ^4^ is defined as the proportion of records of the entire dataset that would not have to be screened after a given value of recall has been achieved and was also calculated at 95% recall (*WSS@95%).* Due to the dependence of the WSS on the number of relevant records, we additionally computed the *normalized WSS* (*n-WSS@95%)* introduced by Kusa *et al.* ^36^ for better comparison between the prognosis and intervention reviews as the latter have fewer relevant records.

The absolute *workload reduction* was defined by both the number of records not needed to screen (assuming the reviewer could stop screening at 95% recall) and by the corresponding amount of time saved in hours. To calculate the time saved, an average duration of 30s was considered as the time needed to screen one record, i.e., title and abstract.

## 3 Results

### 3.1 Screening tool performance in original datasets

**Figure 2:**
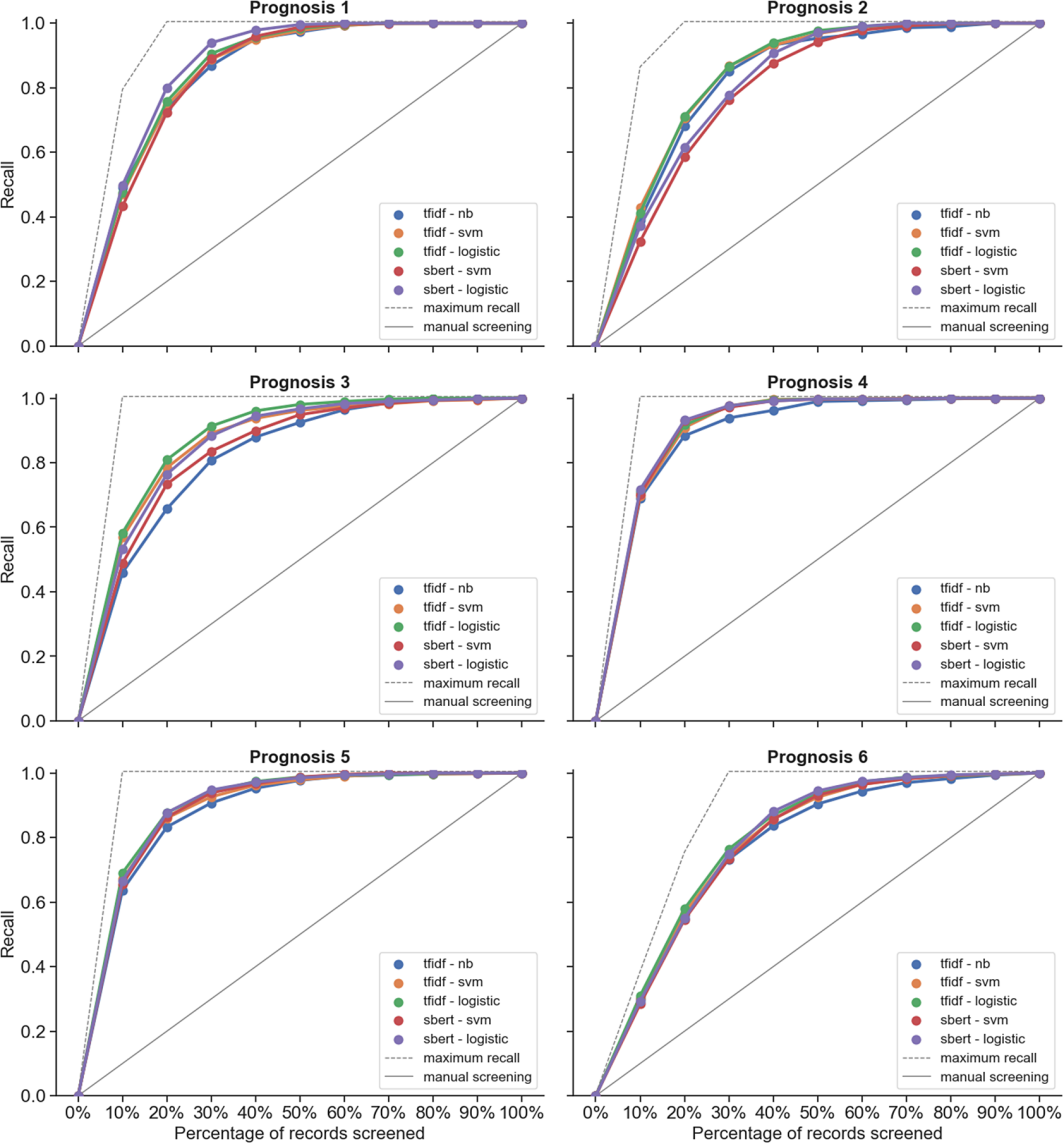
Performance in terms of recall at increasing percentages of records screened for prognosis reviews. Abbreviations: logistic: logistic regression; NB: Naive Bayes; SVM: support vector machine; sBERT: sentence Bidirectional Encoder Representations from Transformers; TF-IDF: term frequency-inverse document frequency.

Based on the original datasets, the intervention reviews (Figure 1) generally reached a recall of 95% earlier in the simulations of the screening process compared to prognosis reviews (Figure 2), that is, a smaller percentage of records needs to be screened for intervention reviews to find 95% of the relevant records. This is also apparent from the higher WSS@95% values that were achieved for intervention reviews (WSS@95% range: 0.613–0.895) compared to prognosis reviews (WSS@95% range: 0.324–0.597), and this difference in WSS between intervention and prognosis reviews remained after normalization of the WSS@95% (*n*-WSS@95% range 0.674–0.946 and 0.454–0.701, respectively) (Part II: Table A1 in the Supplementary Material; default TF-IDF with NB models). This corresponds to a workload reduction of 17.4–76.4 hours (2.087–9.137 records) for intervention reviews compared to a workload reduction of 3.6–54.4 hours (430–6.524 records) for prognosis reviews. However, the precision@95% was overall much lower for intervention reviews (precision range: 0.024–0.057) compared to prognosis reviews (precision range: 0.115–0.400) (Part II: Table A1 in the Supplementary Material; default TF-IDF with NB models).

### 3.2 Variations between prognosis review scopes

**Figure 3:**
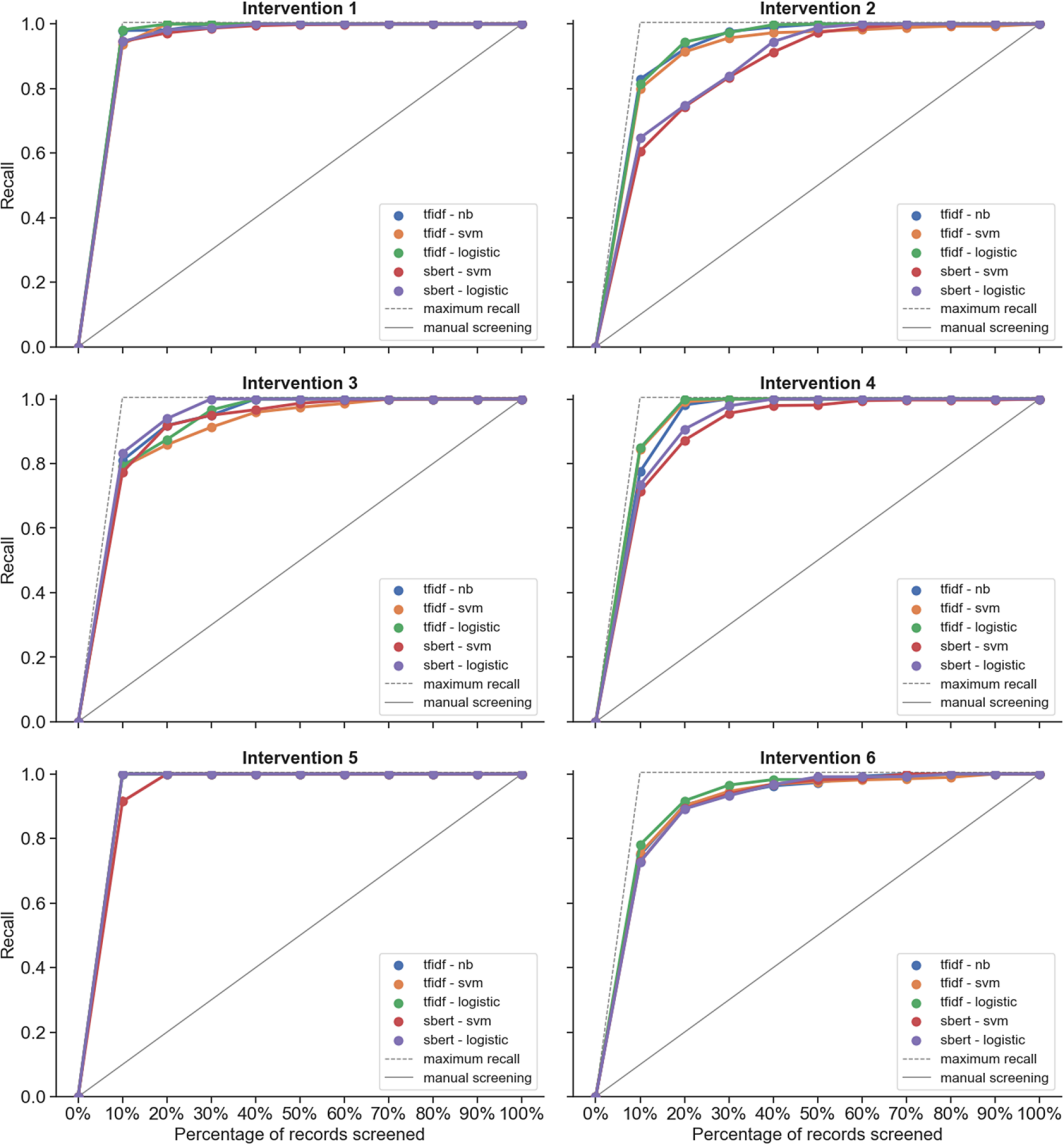
Performance in terms of recall at increasing percentages of records screened for intervention reviews. Abbreviations: logistic: Logistic Regression; NB: Naive Bayes; SVM: Support Vector Machine; sBERT: sentence Bidirectional Encoder Representations from Transformers; TF-IDF: Term Frequency-Inverse Document Frequency.

With regard to the effect of the scope of the prognosis reviews on the performance of the screening tool, there was no indication of increased performance for reviews with narrower scopes (prognosis reviews 6 and 2), compared to moderate scopes (prognosis reviews 4 and 5) or to broad scopes (prognosis reviews 1 and 3) based on the simulations of the original datasets (Figures 2 and 3).

### 3.3 Variations in modeling methods within the screening

The simulations showed that the five modeling methods within the screening tool did not clearly differ in screening performance, except for a slight decreased performance when using sBERT as the feature extraction model in intervention reviews 2 and 4 and in prognosis review 2 compared to using TF-IDF (Figures 1 and 2). These and some slight differences between other models, if present, were usually found earlier in the screening process and would therefore not affect the WSS@95% or precision@95% (Part II: Table A1 in the Supplementary Material). Given this similarity in performance between the models, the remaining results were focused on the default models (TF-IDF with NB) only.

### 3.4 Variations between the number of (relevant) records

When the total number of records and the number of relevant records were equal between intervention and prognosis reviews after manual adaptation (500, 1,000, or 2,000 records with 50 relevant), the performance of the prognosis reviews still did not fully match the intervention reviews (Part II: Figures A2–A7 in the Supplementary Material). The WSS@95% was generally slightly larger for the intervention reviews (WSS@95% range: 0.446–0.865) compared to the prognosis reviews (WSS@95% range 0.355–0.738) and also after normalization (*n*-WSS@95% range: 0.513–0.939 and *n*-WSS@95% range: 0.410–0.801, respectively) (Part III: Table A2 in the Supplementary Material). The precision was also slightly higher for intervention reviews (precision@95% range: 0.041–0.5819) compared to prognosis reviews (precision@95% range: 0.034–0.291) (Part III: Table A2 in the Supplementary Material). In addition to this slight difference in performance between prognosis and intervention reviews, the specific data that was sampled in the adapted datasets (i.e., the fixed numbers of randomly sampled relevant and irrelevant records) also affected the performance of the screening tool (Part III: Figures A2 and A3 in the Supplementary Material).

## 4 Discussion

The performance of AI-based semi-automated title–abstract screening methods was compared between prognosis studies and intervention studies. We selected one of the available screening tools (ASReview) to illustrate the performance of semi-automated title–abstract screening. The amount of work saved, expressed as the (*n*-)WSS@95%, was slightly larger for intervention reviews, but the precision (i.e., positive predictive value) of the tool was lower compared to prognosis reviews. When the proportion of relevant records was manipulated by randomly sampling equal numbers of records for intervention and prognosis review datasets, the WSS@95% was still slightly larger for intervention reviews, and also, the precision was larger compared to prognosis reviews. Furthermore, there were only minor effects of modeling methods in either review type, and there was no clear effect of the scope (i.e., breadth of the research question) of the prognosis review on the performance of the tool.

As would be expected, the screening tool performed slightly worse for prognosis reviews compared to intervention reviews, but this difference was not substantial. It is plausible that this lower performance was due to the larger heterogeneity in abstracts of prognosis studies, causing the feature extraction models to have more difficulty in learning the appropriate features to subsequently classify the abstracts as relevant or irrelevant. Accordingly, the *percentage* of work saved was slightly higher for intervention reviews. However, the *absolute* number of records that needs to be screened is, in practice, generally higher for prognosis reviews. Therefore, this implies that the *absolute* number of records that do not need to be screened, thus the absolute amount of work saved, could in practice be much larger for prognosis reviews. Future work could further evaluate the potential impact of abstract heterogeneity on screening tool performance by incorporating reviews from other domains that may be more affected by this issue.

It should be noted that a considerable part of the initial difference in performance between the original datasets of prognosis and intervention reviews could be explained by the lower number of actually relevant records in the intervention review datasets. A low number of relevant records impacts the maximum achievable values of the performance metrics (i.e., these values are increased for WSS and recall and decreased for precision), which was compensated for in this study by our selection of intervention and prognosis reviews based on similar number of records and by the additional analysis in which equal numbers of (included) records were selected from the reviews for simulations. Consequently, this highlights that evaluating performance on datasets with limited variation in numbers of relevant records and with limited reporting of metrics may give misleading impressions on the general performance of a screening tool. The same applies to the size of the dataset; the maximum achievable value for WSS is inherently related to the total number of records (under a constant number of relevant records). Nevertheless, these aspects are hardly ever addressed in comparative evaluation or validation studies of semi-automated title–abstract screening tools but should be considered along with the need for standardized evaluation and reporting.

Interestingly, the performance of the tool did not differ between the size of the scope of the prognosis reviews. Nevertheless, there were only a few reviews for each scope breadth, and the true relation between scope breadth and coherence of the relevant abstracts was not further assessed in this study, but could influence the model performance.^14^ Furthermore, similar to the results of the comparative review on screening tool performance by Feng *et al.* ^10^ there were only minor differences in performance between the modeling methods. These differences mainly involved the sBERT feature extraction model which not only took longer to train due to its complexity, but also performed similar to even slightly worse compared to the simple method of TF-IDF as has also previously been suggested.^37^

Even though this study indicates that the performance of semi-automated screening tools is not substantially different between SRs of prognosis and intervention studies, there are still some issues that need to be overcome before the implementation of semi-automated tools in either review type. First, there is still a lack of trust by the SR community in the adoption of these tools^38^ which could be related to the lack of standardized evaluation and (external) validation of tools.^39^ Without standardized ways of evaluation of tool performance, it becomes difficult to compare tools and to draw conclusions on the generalizability of performance to other contexts. Although there is a lack of validation studies, the number of studies on developing new tools or algorithms is growing,^5^ making end users lose track of what tools they can use for their specific context.

Furthermore, there is still no consensus reached on what the appropriate stopping criterion for semi-automated screening should be in order to balance the trade-off between screening costs and sensitivity of the classification, including how this can prospectively be defined with sufficient certainty. As a result, such stopping criteria have not been incorporated in most of the currently available ready-to-use tools.^37^ Stopping criteria that have been proposed include time-based or pragmatic approaches (i.e., stopping after a certain time or percentage screened), heuristic or data-driven approaches (i.e., stopping after encountering a predefined number of consecutive irrelevant records), and sampling approaches (i.e., estimating the number of relevant records based on screening a sample of the records). More statistical methods have also been proposed which use more advanced methods to prospectively estimate the recall including its certainty.^40^^,^ ^41^

Limitations of the current study include the reference standard of manual screening that was used in the simulations. This standard is likely to contain errors due to screening fatigue and screeners’ over-inclusiveness.^5^ Since this effect would be the same for the intervention and prognosis datasets, it could be assumed to not have affected the comparison between the two review types. The datasets consisted of a convenience sample and are thereby no true representation of all reviews of either type which could possibly have led to selection bias. The records eligible for full text screening were used as the reference standard as opposed to records that were final inclusions in the review, in order to have the simulations resemble title–abstract screening in practice. As such, we have no clear indication of the impact of the missed records during title–abstract screening on missing final inclusions in the review and subsequently on potential bias in the results.

## 5 Conclusion

In this study, we evaluated the performance of an AI-based screening tool for semi-automated title–abstract screening in the development of SRs for both intervention and prognosis studies. While intervention reviews have been extensively studied in the context of screening tool evaluations, prognosis reviews have not yet been thoroughly assessed. Despite the anticipated lower performance for prognosis reviews due to the larger heterogeneity of abstracts, the screening tool still demonstrated significant amounts of workload reduction for prognosis reviews that were only slightly lower compared to intervention reviews. Furthermore, we found no significant effects of the review’s scope (i.e., breadth of the research question of the review) or the specific modeling methods applied within the tool on its performance for either type of review. Instead, factors that did significantly affect screening tool performance were related to the number of records retrieved and the proportion of relevant records within the review. Given these findings, further evaluation studies should focus on developing methods to predict the tool’s performance based on the number of records retrieved and the expected proportion of relevant records. Nevertheless, the implementation of a screening tool in which active learning is combined with machine learning algorithms has been shown to be sufficiently applicable for semi-automated screening in both intervention and prognosis SRs.

## Supporting information

Spiero et al. supplementary material Spiero et al. supplementary material

## Data Availability

The code and data of our study are available at https://github.com/isa-sp/SR_semi-automated_screening. The data were partially derived from the publicly available data from the CLEF 2019 eHealth task at https://github.com/CLEF-TAR/tar/.

## Supplementary material

To view supplementary material for this article, please visit http://doi.org/10.1017/rsm.2025.10025.
