# Supplementary material for: Evaluation of semi-automated record screening methods for systematic reviews of prognosis studies and intervention studies
Source: Res Synth Methods. 2025 Jul 22;16(6):975–89. doi: 10.1017/rsm.2025.10025 (PMC12657655; doi:10.1017/rsm.2025.10025)
Supplement: Spiero et al. supplementary material [file S1759287925100252sup001.pdf]

Appendix

Part I: Calculation of performance metrics

- **Recall (or sensitivity)**: the proportion of correctly classified positives by the tool among the total number of positives. This is calculated by the true positives (*TP*) and false negatives (*FN*) at a given number of records screened (*n*).

$$recall_{at\ n\ records\ screened} = \frac{TP}{TP + FN} \tag{1}$$

- **Maximum achievable recall**: the maximum proportion of correctly classified positives that could in theory be achieved. We calculated this by using the highest number of true positives (max *TP*) and the lowest number of false negatives (min *FN*) at a given number of records screened (*n*) that would be possible based on the number of relevant records (*I*) that are present in the dataset.

$$\max(TP)_{at\ n\ records\ screened} = \frac{1}{2}\left((n + I) - |\ n - I\ |\right) \tag{2}$$

$$\min(FN)_{at\ n\ records\ screened} = \frac{1}{2}\left(|\ I - n\ | + (I - n)\right) \tag{3}$$

$$\max(recall)_{at\ n\ records\ screened} = \frac{\max(TP)_{at\ n\ records\ screened}}{\max(TP)_{at\ n\ records\ screened} + \min(FN)_{at\ n\ records\ screened}} = \frac{\frac{1}{2}\left((n+I) - |\ n - I\ |\right)}{\frac{1}{2}\left((n+I) - |\ n - I\ |\right) + \frac{1}{2}\left(|\ I - n\ | + (I - n)\right)} \tag{4}$$

- **Precision (or positive predictive value)**: the proportion of correctly classified positives (*TP*) among the total number of classified positives (true positives (*TP*) plus false positives (*FP*)). This is calculated at the point at which screening reached 95% recall, ***precision@95%***.

$$precision@95\% = \frac{TP}{TP + FP} \tag{5}$$

- **Work Saved over Sampling (*WSS*)**: introduced by Cohen *et al.* [4] and defined as the proportion of records of the entire dataset (*N*) that will not have to be screened (the true negatives (*TN*) and false negatives (*FN*)) when a give value of recall has been achieved, usually also at 95% recall, ***WSS@95%***.

$$WSS@95\% = (TN + FN)\ /\ N - (1.0 - 0.95) \tag{6}$$

- **Normalized WSS (*n-WSS*)**: introduced by Kusa *et al.* [39] is defined as the WSS in which the value is compensated by the number of relevant records (I) and irrelevant records (E) in the entire dataset of records (N). It is also calculated at 95% recall; ***n-WSS@95%.***

$$\max(WSS@95\%) = \frac{E+(I\cdot(1-0.95))}{N} - (1-0.95) \tag{7}$$

$$\min(WSS@95\%) = \frac{0+(I\cdot(1-0.95))}{N} - (1-0.95) \tag{8}$$

$$nWSS@95\% = \frac{WSS@95\% - \min(WSS@95\%)}{\max(WSS@95\%) - \min(WSS@95\%)} \tag{9}$$

Part II: Results of the simulations with the original review datasets

Table A1 | The raw and normalized Work Saved over Sampling at 95% recall (WSS@95% and n-WSS@95%), the precision at 95% recall (precision@95%) and the workload reduction for each combination of review and modelling methods and averaged over 200 simulations

Review	Train model	Feature model	WSS@95% (CI)	n-WSS@95% (CI)	Precision@95% (CI)	Workload reduction in record numbers (CI)	Workload reduction in hours (CI)
**Prognosis reviews**							
Prog1	logistic	sbert	0.635 (0.633-0.636)	0.770 (0.769-0.772)	0.368 (0.367-0.369)	1,699 (1696-1703)	14.2 (14.1-14.2)
	logistic	tfidf	0.573 (0.571-0.575)	0.700 (0.698-0.703)	0.309 (0.308-0.311)	1,547 (1542-1552)	12.9 (12.8-12.9)
	nb	tfidf	0.554 (0.553-0.555)	0.678 (0.677-0.679)	0.292 (0.292-0.293)	1,499 (1497-1502)	12.5 (12.5-12.5)
	svm	sbert	0.574 (0.572-0.576)	0.700 (0.698-0.703)	0.310 (0.308-0.311)	1,548 (1543-1553)	12.9 (12.9-12.9)
	svm	tfidf	0.549 (0.547-0.552)	0.673 (0.670-0.675)	0.288 (0.287-0.289)	1,488 (1482-1493)	12.4 (12.4-12.4)
Prog2	logistic	sbert	0.506 (0.503-0.509)	0.603 (0.600-0.607)	0.221 (0.220-0.222)	432 (430-434)	3.6 (3.6-3.6)
	logistic	tfidf	0.541 (0.536-0.545)	0.644 (0.639-0.649)	0.237 (0.235-0.239)	459 (456-463)	3.8 (3.8-3.9)
	nb	tfidf	0.503 (0.497-0.509)	0.600 (0.593-0.607)	0.220 (0.217-0.222)	430 (425-434)	3.6 (3.5-3.6)
	svm	sbert	0.443 (0.438-0.447)	0.531 (0.526-0.536)	0.189 (0.188-0.190)	383 (379-386)	3.2 (3.2-3.2)
	svm	tfidf	0.513 (0.508-0.518)	0.612 (0.606-0.618)	0.230 (0.227-0.232)	437 (433-441)	3.6 (3.6-3.7)
Prog3	logistic	sbert	0.530 (0.529-0.532)	0.618 (0.616-0.621)	0.156 (0.155-0.156)	2,827 (2818-2837)	23.6 (23.5-23.6)
	logistic	tfidf	0.575 (0.573-0.577)	0.666 (0.664-0.668)	0.176 (0.175-0.177)	3,044 (3035-3053)	25.4 (25.3-25.4)
	nb	tfidf	0.378 (0.376-0.380)	0.454 (0.452-0.456)	0.115 (0.115-0.115)	2,085 (2077-2092)	17.4 (17.3-17.4)
	svm	sbert	0.441 (0.439-0.444)	0.522 (0.519-0.525)	0.129 (0.128-0.129)	2,394 (2380-2407)	19.9 (19.8-20.1)
	svm	tfidf	0.499 (0.496-0.503)	0.585 (0.581-0.588)	0.148 (0.147-0.149)	2,675 (2658-2692)	22.3 (22.1-22.4)
Prog4	logistic	sbert	0.717 (0.716-0.719)	0.834 (0.833-0.835)	0.352 (0.350-0.353)	3,280 (3276-3285)	27.3 (27.3-27.4)
	logistic	tfidf	0.707 (0.706-0.708)	0.823 (0.821-0.824)	0.335 (0.334-0.336)	3,236 (3231-3241)	27.0 (26.9-27.0)
	nb	tfidf	0.597 (0.595-0.599)	0.701 (0.699-0.703)	0.231 (0.230-0.233)	2,764 (2755-2772)	23.0 (23.0-23.1)
	svm	sbert	0.710 (0.709-0.712)	0.826 (0.824-0.828)	0.341 (0.339-0.343)	3,249 (3242-3256)	27.1 (27.0-27.1)
	svm	tfidf	0.696 (0.694-0.697)	0.810 (0.809-0.812)	0.322 (0.320-0.323)	3,188 (3181-3194)	26.6 (26.5-26.6)
Prog5	logistic	sbert	0.644 (0.644-0.645)	0.757 (0.756-0.757)	0.275 (0.274-0.275)	7,406 (7398-7413)	61.7 (61.6-61.8)
	logistic	tfidf	0.632 (0.631-0.632)	0.742 (0.741-0.743)	0.264 (0.263-0.264)	7,268 (7259-7276)	60.6 (60.5-60.6)
	nb	tfidf	0.562 (0.561-0.563)	0.666 (0.665-0.667)	0.216 (0.216-0.217)	6,524 (6515-6533)	54.4 (54.3-54.4)
	svm	sbert	0.619 (0.618-0.621)	0.729 (0.728-0.730)	0.255 (0.254-0.256)	7,138 (7126-7151)	59.5 (59.4-59.6)
	svm	tfidf	0.595 (0.594-0.596)	0.702 (0.701-0.703)	0.237 (0.236-0.238)	6,877 (6863-6890)	57.3 (57.2-57.4)
Prog6	logistic	sbert	0.432 (0.431-0.432)	0.634 (0.633-0.635)	0.482 (0.482-0.483)	1,926 (1922-1929)	16.0 (16.0-16.1)
	logistic	tfidf	0.424 (0.423-0.425)	0.623 (0.622-0.624)	0.476 (0.476-0.477)	1,894 (1891-1898)	15.8 (15.8-15.8)
	nb	tfidf	0.324 (0.323-0.325)	0.487 (0.485-0.488)	0.400 (0.400-0.400)	1,495 (1492-1498)	12.5 (12.4-12.5)
	svm	sbert	0.409 (0.408-0.410)	0.603 (0.601-0.604)	0.464 (0.464-0.465)	1,835 (1831-1840)	15.3 (15.3-15.3)
	svm	tfidf	0.397 (0.396-0.398)	0.586 (0.585-0.588)	0.452 (0.451-0.453)	1,787 (1783-1791)	14.9 (14.9-14.9)
**Intervention reviews**							
Int1	logistic	sbert	0.853 (0.848-0.858)	0.907 (0.902-0.912)	0.058 (0.053-0.062)	8,273 (8227-8319)	68.9 (68.6-69.3)
	logistic	tfidf	0.866 (0.864-0.867)	0.920 (0.918-0.921)	0.054 (0.054-0.055)	8,386 (8374-8398)	69.9 (69.8-70.0)
	nb	tfidf	0.863 (0.863-0.864)	0.917 (0.917-0.918)	0.054 (0.053-0.054)	8,366 (8359-8374)	69.7 (69.7-69.8)
	svm	sbert	0.844 (0.839-0.850)	0.898 (0.893-0.904)	0.051 (0.048-0.054)	8,193 (8144-8243)	68.3 (67.9-68.7)
	svm	tfidf	0.845 (0.843-0.847)	0.899 (0.897-0.901)	0.043 (0.042-0.044)	8,196 (8178-8215)	68.3 (68.1-68.5)
Int2	logistic	sbert	0.535 (0.531-0.538)	0.588 (0.584-0.591)	0.015 (0.015-0.015)	7,203 (7159-7247)	60.0 (59.7-60.4)

Review	Train model	Feature model	WSS@95% (CI)	n-WSS@95% (CI)	Precision@95% (CI)	Workload reduction in record numbers (CI)	Workload reduction in hours (CI)
	logistic	tfidf	0.732 (0.730-0.734)	0.787 (0.785-0.789)	0.028 (0.028-0.028)	9,637 (9613-9662)	80.3 (80.1-80.5)
	nb	tfidf	0.695 (0.691-0.698)	0.749 (0.745-0.753)	0.024 (0.024-0.024)	9,173 (9126-9219)	76.4 (76.0-76.8)
	svm	sbert	0.508 (0.502-0.515)	0.561 (0.554-0.567)	0.014 (0.014-0.014)	6,877 (6798-6957)	57.3 (56.6-58.0)
	svm	tfidf	0.659 (0.652-0.667)	0.713 (0.706-0.720)	0.021 (0.021-0.021)	8,736 (8645-8828)	72.8 (72.0-73.6)
Int3	logistic	sbert	0.735 (0.728-0.742)	0.791 (0.784-0.798)	0.046 (0.045-0.048)	2,210 (2191-2229)	18.4 (18.3-18.6)
	logistic	tfidf	0.681 (0.677-0.685)	0.736 (0.731-0.740)	0.035 (0.034-0.035)	2,058 (2046-2070)	17.1 (17.0-17.2)
	nb	tfidf	0.691 (0.683-0.700)	0.746 (0.738-0.755)	0.038 (0.036-0.039)	2,087 (2064-2110)	17.4 (17.2-17.6)
	svm	sbert	0.624 (0.608-0.640)	0.678 (0.661-0.695)	0.032 (0.031-0.034)	1,898 (1851-1944)	15.8 (15.4-16.2)
	svm	tfidf	0.589 (0.581-0.598)	0.642 (0.634-0.651)	0.026 (0.026-0.027)	1,799 (1775-1823)	15.0 (14.8-15.2)
Int4	logistic	sbert	0.716 (0.712-0.720)	0.772 (0.768-0.776)	0.045 (0.044-0.046)	2,966 (2951-2982)	24.7 (24.6-24.9)
	logistic	tfidf	0.815 (0.813-0.818)	0.873 (0.871-0.876)	0.077 (0.076-0.078)	3,352 (3342-3361)	27.9 (27.8-28.0)
	nb	tfidf	0.775 (0.773-0.777)	0.833 (0.831-0.834)	0.057 (0.056-0.057)	3,196 (3189-3203)	26.6 (26.6-26.7)
	svm	sbert	0.679 (0.674-0.683)	0.735 (0.730-0.739)	0.038 (0.037-0.038)	2,824 (2806-2841)	23.5 (23.4-23.7)
	svm	tfidf	0.812 (0.809-0.814)	0.870 (0.868-0.872)	0.073 (0.072-0.074)	3,338 (3329-3347)	27.8 (27.7-27.9)
Int5	logistic	sbert	0.900 (0.898-0.901)	0.951 (0.950-0.952)	0.024 (0.023-0.024)	8,422 (8410-8434)	70.2 (70.1-70.3)
	logistic	tfidf	0.889 (0.888-0.891)	0.941 (0.939-0.942)	0.021 (0.021-0.021)	8,330 (8316-8343)	69.4 (69.3-69.5)
	nb	tfidf	0.895 (0.894-0.896)	0.946 (0.945-0.947)	0.024 (0.024-0.024)	8,381 (8371-8390)	69.8 (69.8-69.9)
	svm	sbert	0.837 (0.833-0.842)	0.888 (0.884-0.893)	0.011 (0.011-0.012)	7,869 (7828-7909)	65.6 (65.2-65.9)
	svm	tfidf	0.886 (0.884-0.888)	0.937 (0.936-0.939)	0.017 (0.016-0.017)	8,301 (8286-8317)	69.2 (69.0-69.3)
Int6	logistic	sbert	0.614 (0.611-0.618)	0.675 (0.671-0.679)	0.054 (0.053-0.054)	3,583 (3562-3604)	29.9 (29.7-30.0)
	logistic	tfidf	0.706 (0.703-0.708)	0.768 (0.766-0.771)	0.073 (0.072-0.073)	4,074 (4062-4087)	34.0 (33.8-34.1)
	nb	tfidf	0.613 (0.609-0.618)	0.674 (0.669-0.678)	0.053 (0.053-0.054)	3,576 (3552-3600)	29.8 (29.6-30.0)
	svm	sbert	0.630 (0.626-0.634)	0.691 (0.687-0.695)	0.056 (0.056-0.057)	3,666 (3643-3689)	30.6 (30.4-30.7)
	svm	tfidf	0.637 (0.630-0.644)	0.698 (0.691-0.706)	0.058 (0.057-0.059)	3,705 (3665-3745)	30.9 (30.5-31.2)

Abbreviations: (n-)WSS: (normalized-)Work Saved over Sampling; CI: Confidence Interval; logistic: Logistic Regression; nb: Naive Bayes; SVM: Support Vector Machine; sbert: sentence Bidirectional Encoder Representations from Transformers; tfidf: Term Frequency-Inverse Document Frequency.

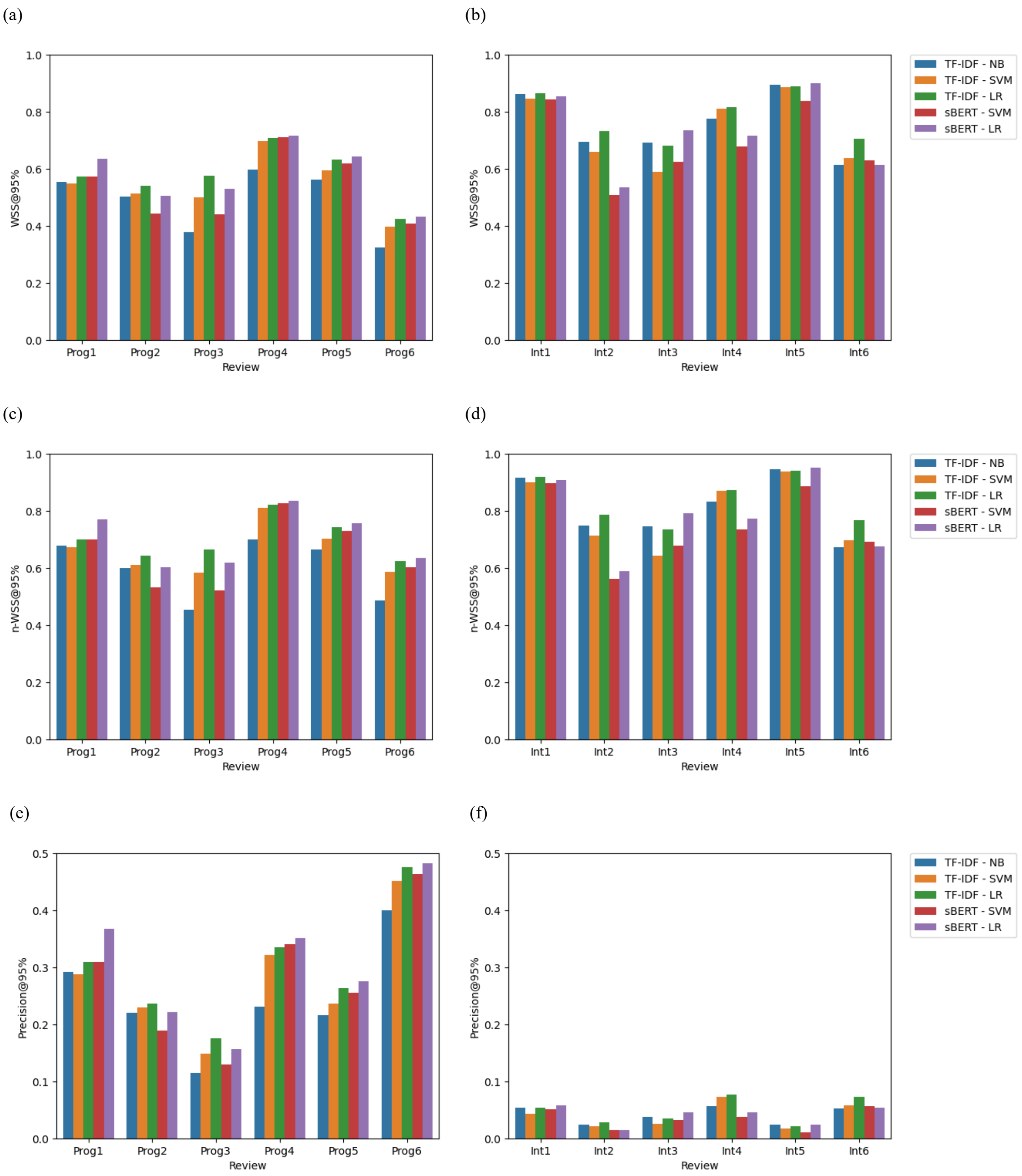

Figure A1 | Performance metrics for the simulations with the intervention reviews and prognosis reviews for five different combinations of feature extraction and classification models: (a) WSS@95% for the intervention reviews (b) WSS@95% for the prognosis reviews, (c) n-WSS@95% for the intervention reviews, (d) n-WSS@95% for the intervention reviews, (e) precision@95% for the intervention reviews, and (f) precision@95% for the prognosis reviews. Abbreviations: (n-)WSS: (normalized-)Work Saved over Sampling; logistic: Logistic Regression; nb: Naive Bayes; SVM: Support Vector Machine; sbert: sentence Bidirectional Encoder Representations from Transformers; tfidf: Term Frequency-Inverse Document Frequency.

Part III: Results of the simulations with manually adapted review datasets

Table A2 | The raw and normalized Work –Saved over Sampling at 95% recall (WSS@95% and n-WSS@95%), the precision at 95% recall (precision@95%), and the workload reduction for the intervention and prognosis reviews with manually adapted numbers of records and percentages of relevant records (each sampled 5 times from the original dataset; indicated with seed number). Each dataset was simulated 200 times with the default NB and TF-IDF models and the average values are presented.

Review	Seed	Total records	Relevant records	WSS@95% (CI)	n-WSS@95% (CI)	Precision@95% (CI)	Workload reduction in record numbers (CI)	Workload reduction in hours (CI)
**Prognosis reviews**								
Prog3	1	500	0.1	0.439 (0.434-0.444)	0.506 (0.500-0.511)	0.143 (0.142-0.145)	244 (242-247)	2.0 (2.0-2.1)
	2	500	0.1	0.532 (0.527-0.536)	0.611 (0.606-0.616)	0.178 (0.177-0.180)	291 (289-293)	2.4 (2.4-2.4)
	3	500	0.1	0.393 (0.386-0.400)	0.453 (0.445-0.461)	0.136 (0.135-0.138)	221 (218-225)	1.8 (1.8-1.9)
	4	500	0.1	0.530 (0.524-0.535)	0.609 (0.602-0.615)	0.179 (0.176-0.181)	290 (287-293)	2.4 (2.4-2.4)
	5	500	0.1	0.355 (0.347-0.362)	0.410 (0.401-0.418)	0.126 (0.125-0.127)	202 (199-206)	1.7 (1.7-1.7)
Prog3	1	1000	0.05	0.438 (0.432-0.443)	0.496 (0.490-0.501)	0.073 (0.072-0.073)	488 (482-493)	4.1 (4.0-4.1)
	2	1000	0.05	0.582 (0.579-0.585)	0.649 (0.646-0.652)	0.098 (0.098-0.099)	632 (629-635)	5.3 (5.2-5.3)
	3	1000	0.05	0.397 (0.390-0.405)	0.453 (0.444-0.461)	0.068 (0.067-0.069)	447 (440-455)	3.7 (3.7-3.8)
	4	1000	0.05	0.556 (0.551-0.561)	0.622 (0.616-0.627)	0.099 (0.098-0.100)	606 (601-611)	5.1 (5.0-5.1)
	5	1000	0.05	0.364 (0.355-0.373)	0.417 (0.407-0.426)	0.064 (0.063-0.065)	414 (405-423)	3.4 (3.4-3.5)
Prog3	1	2000	0.025	0.429 (0.423-0.435)	0.483 (0.477-0.488)	0.035 (0.035-0.035)	958 (947-970)	8.0 (7.9-8.1)
	2	2000	0.025	0.610 (0.607-0.613)	0.669 (0.666-0.673)	0.055 (0.055-0.056)	1,320 (1313-1327)	11.0 (10.9-11.1)
	3	2000	0.025	0.405 (0.398-0.412)	0.458 (0.450-0.465)	0.034 (0.034-0.034)	910 (896-923)	7.6 (7.5-7.7)
	4	2000	0.025	0.584 (0.580-0.588)	0.642 (0.638-0.647)	0.051 (0.051-0.052)	1,268 (1260-1277)	10.6 (10.5-10.6)
	5	2000	0.025	0.401 (0.392-0.410)	0.454 (0.445-0.463)	0.034 (0.033-0.034)	902 (884-919)	7.5 (7.4-7.7)
Prog4	1	500	0.1	0.602 (0.595-0.609)	0.691 (0.683-0.699)	0.220 (0.215-0.224)	326 (323-330)	2.7 (2.7-2.7)
	2	500	0.1	0.653 (0.642-0.664)	0.749 (0.736-0.761)	0.267 (0.258-0.277)	351 (346-357)	2.9 (2.9-3.0)
	3	500	0.1	0.599 (0.593-0.606)	0.688 (0.680-0.695)	0.212 (0.208-0.215)	325 (321-328)	2.7 (2.7-2.7)
	4	500	0.1	0.605 (0.600-0.610)	0.695 (0.689-0.700)	0.223 (0.219-0.226)	328 (325-330)	2.7 (2.7-2.8)
	5	500	0.1	0.683 (0.679-0.686)	0.783 (0.779-0.787)	0.291 (0.288-0.294)	366 (365-368)	3.1 (3.0-3.1)
Prog4	1	1000	0.05	0.630 (0.625-0.635)	0.700 (0.695-0.706)	0.117 (0.115-0.119)	680 (675-685)	5.7 (5.6-5.7)
	2	1000	0.05	0.663 (0.649-0.678)	0.736 (0.720-0.751)	0.145 (0.138-0.152)	713 (699-728)	5.9 (5.8-6.1)
	3	1000	0.05	0.581 (0.573-0.589)	0.648 (0.640-0.657)	0.102 (0.100-0.105)	631 (623-639)	5.3 (5.2-5.3)
	4	1000	0.05	0.612 (0.605-0.618)	0.681 (0.674-0.688)	0.116 (0.113-0.118)	662 (655-668)	5.5 (5.5-5.6)
	5	1000	0.05	0.698 (0.694-0.701)	0.772 (0.769-0.775)	0.150 (0.149-0.152)	748 (744-751)	6.2 (6.2-6.3)
Prog4	1	2000	0.025	0.603 (0.599-0.608)	0.662 (0.657-0.667)	0.054 (0.054-0.055)	1,307 (1297-1316)	10.9 (10.8-11.0)
	2	2000	0.025	0.658 (0.643-0.673)	0.719 (0.703-0.734)	0.072 (0.069-0.076)	1,416 (1387-1445)	11.8 (11.6-12.0)
	3	2000	0.025	0.590 (0.582-0.597)	0.648 (0.640-0.656)	0.053 (0.051-0.054)	1,280 (1264-1295)	10.7 (10.5-10.8)
	4	2000	0.025	0.604 (0.597-0.611)	0.663 (0.655-0.670)	0.057 (0.055-0.058)	1,308 (1294-1322)	10.9 (10.8-11.0)
	5	2000	0.025	0.711 (0.707-0.714)	0.773 (0.769-0.777)	0.080 (0.079-0.082)	1,521 (1513-1529)	12.7 (12.6-12.7)
Prog5	1	500	0.1	0.440 (0.430-0.450)	0.507 (0.496-0.518)	0.147 (0.144-0.150)	245 (240-250)	2.0 (2.0-2.1)
	2	500	0.1	0.421 (0.413-0.429)	0.485 (0.476-0.495)	0.140 (0.139-0.142)	236 (231-240)	2.0 (1.9-2.0)
	3	500	0.1	0.551 (0.546-0.556)	0.633 (0.627-0.638)	0.184 (0.182-0.185)	300 (298-303)	2.5 (2.5-2.5)
	4	500	0.1	0.589 (0.586-0.592)	0.676 (0.672-0.680)	0.213 (0.211-0.214)	319 (318-321)	2.7 (2.6-2.7)
	5	500	0.1	0.674 (0.670-0.677)	0.772 (0.769-0.776)	0.268 (0.265-0.271)	362 (360-363)	3.0 (3.0-3.0)

Review	Seed	Total records	Relevant records	WSS@95% (CI)	n-WSS@95% (CI)	Precision@95% (CI)	Workload reduction in record numbers (CI)	Workload reduction in hours (CI)
Prog5	1	1000	0.05	0.459 (0.450-0.467)	0.518 (0.508-0.527)	0.075 (0.073-0.077)	508 (500-517)	4.2 (4.2-4.3)
	2	1000	0.05	0.421 (0.414-0.428)	0.478 (0.470-0.486)	0.069 (0.068-0.070)	471 (464-478)	3.9 (3.9-4.0)
	3	1000	0.05	0.564 (0.560-0.569)	0.630 (0.625-0.635)	0.097 (0.096-0.097)	614 (610-619)	5.1 (5.1-5.2)
	4	1000	0.05	0.621 (0.618-0.624)	0.691 (0.688-0.694)	0.115 (0.115-0.116)	671 (668-674)	5.6 (5.6-5.6)
	5	1000	0.05	0.723 (0.720-0.726)	0.799 (0.796-0.802)	0.163 (0.161-0.164)	773 (770-776)	6.4 (6.4-6.5)
Prog5	1	2000	0.025	0.478 (0.470-0.486)	0.533 (0.525-0.541)	0.040 (0.039-0.041)	1,056 (1040-1072)	8.8 (8.7-8.9)
	2	2000	0.025	0.451 (0.446-0.455)	0.505 (0.500-0.510)	0.037 (0.037-0.037)	1,001 (992-1011)	8.3 (8.3-8.4)
	3	2000	0.025	0.596 (0.592-0.599)	0.654 (0.651-0.658)	0.051 (0.051-0.052)	1,292 (1284-1299)	10.8 (10.7-10.8)
	4	2000	0.025	0.653 (0.651-0.655)	0.714 (0.711-0.716)	0.063 (0.063-0.063)	1,406 (1402-1410)	11.7 (11.7-11.8)
	5	2000	0.025	0.738 (0.736-0.741)	0.801 (0.799-0.804)	0.087 (0.086-0.087)	1,577 (1572-1581)	13.1 (13.1-13.2)
Prog6	1	500	0.1	0.450 (0.430-0.469)	0.518 (0.495-0.540)	0.165 (0.158-0.171)	250 (240-260)	2.1 (2.0-2.2)
	2	500	0.1	0.443 (0.435-0.451)	0.510 (0.501-0.519)	0.146 (0.145-0.148)	246 (242-250)	2.1 (2.0-2.1)
	3	500	0.1	0.613 (0.609-0.616)	0.703 (0.699-0.707)	0.222 (0.220-0.224)	331 (330-333)	2.8 (2.7-2.8)
	4	500	0.1	0.504 (0.500-0.509)	0.580 (0.575-0.585)	0.168 (0.167-0.169)	277 (275-280)	2.3 (2.3-2.3)
	5	500	0.1	0.465 (0.459-0.470)	0.535 (0.528-0.541)	0.160 (0.159-0.162)	257 (254-260)	2.1 (2.1-2.2)
Prog6	1	1000	0.05	0.472 (0.455-0.489)	0.532 (0.514-0.550)	0.084 (0.081-0.087)	522 (505-539)	4.3 (4.2-4.5)
	2	1000	0.05	0.453 (0.447-0.459)	0.511 (0.505-0.518)	0.075 (0.074-0.075)	503 (497-509)	4.2 (4.1-4.2)
	3	1000	0.05	0.664 (0.660-0.667)	0.736 (0.732-0.739)	0.131 (0.130-0.132)	714 (710-717)	5.9 (5.9-6.0)
	4	1000	0.05	0.495 (0.489-0.501)	0.557 (0.551-0.563)	0.084 (0.083-0.085)	545 (539-551)	4.5 (4.5-4.6)
	5	1000	0.05	0.465 (0.459-0.471)	0.524 (0.518-0.531)	0.076 (0.075-0.077)	515 (509-521)	4.3 (4.2-4.3)
Prog6	1	2000	0.025	0.465 (0.449-0.481)	0.519 (0.503-0.536)	0.041 (0.040-0.042)	1,030 (998-1062)	8.6 (8.3-8.8)
	2	2000	0.025	0.497 (0.493-0.501)	0.552 (0.548-0.557)	0.041 (0.041-0.041)	1,094 (1085-1102)	9.1 (9.0-9.2)
	3	2000	0.025	0.692 (0.689-0.695)	0.753 (0.750-0.756)	0.072 (0.071-0.073)	1,484 (1478-1489)	12.4 (12.3-12.4)
	4	2000	0.025	0.467 (0.461-0.473)	0.522 (0.516-0.528)	0.040 (0.039-0.040)	1,035 (1023-1046)	8.6 (8.5-8.7)
	5	2000	0.025	0.490 (0.485-0.496)	0.546 (0.540-0.552)	0.041 (0.040-0.041)	1,081 (1069-1092)	9.0 (8.9-9.1)
Prog6	1	500	0.1	0.450 (0.430-0.469)	0.518 (0.495-0.540)	0.165 (0.158-0.171)	250 (240-260)	2.1 (2.0-2.2)
	2	500	0.1	0.443 (0.435-0.451)	0.510 (0.501-0.519)	0.146 (0.145-0.148)	246 (242-250)	2.1 (2.0-2.1)
	3	500	0.1	0.613 (0.609-0.616)	0.703 (0.699-0.707)	0.222 (0.220-0.224)	331 (330-333)	2.8 (2.7-2.8)
	4	500	0.1	0.504 (0.500-0.509)	0.580 (0.575-0.585)	0.168 (0.167-0.169)	277 (275-280)	2.3 (2.3-2.3)
	5	500	0.1	0.465 (0.459-0.470)	0.535 (0.528-0.541)	0.160 (0.159-0.162)	257 (254-260)	2.1 (2.1-2.2)
**Intervention reviews**								
Int1	1	500	0.1	0.820 (0.819-0.822)	0.939 (0.938-0.941)	0.581 (0.577-0.586)	435 (435-436)	3.6 (3.6-3.6)
	2	500	0.1	0.791 (0.789-0.793)	0.906 (0.904-0.908)	0.479 (0.475-0.483)	421 (420-422)	3.5 (3.5-3.5)
	3	500	0.1	0.817 (0.816-0.818)	0.935 (0.934-0.937)	0.562 (0.557-0.568)	434 (433-434)	3.6 (3.6-3.6)
	4	500	0.1	0.808 (0.806-0.809)	0.925 (0.923-0.927)	0.523 (0.519-0.527)	429 (428-430)	3.6 (3.6-3.6)
	5	500	0.1	0.779 (0.777-0.781)	0.892 (0.889-0.894)	0.431 (0.428-0.435)	414 (413-415)	3.5 (3.4-3.5)
Int1	1	1000	0.05	0.840 (0.838-0.842)	0.923 (0.921-0.926)	0.350 (0.344-0.357)	890 (888-892)	7.4 (7.4-7.4)
	2	1000	0.05	0.827 (0.825-0.829)	0.910 (0.908-0.912)	0.313 (0.309-0.317)	877 (875-879)	7.3 (7.3-7.3)
	3	1000	0.05	0.843 (0.841-0.845)	0.927 (0.925-0.929)	0.354 (0.349-0.360)	893 (891-895)	7.4 (7.4-7.5)
	4	1000	0.05	0.833 (0.830-0.835)	0.916 (0.913-0.918)	0.326 (0.321-0.332)	883 (880-885)	7.4 (7.3-7.4)
	5	1000	0.05	0.809 (0.807-0.812)	0.891 (0.888-0.893)	0.269 (0.264-0.274)	859 (857-862)	7.2 (7.1-7.2)
Int1	1	2000	0.025	0.865 (0.863-0.867)	0.932 (0.930-0.934)	0.227 (0.222-0.231)	1,830 (1827-1834)	15.3 (15.2-15.3)
	2	2000	0.025	0.840 (0.839-0.842)	0.907 (0.905-0.908)	0.176 (0.173-0.179)	1,781 (1777-1784)	14.8 (14.8-14.9)
	3	2000	0.025	0.855 (0.853-0.856)	0.921 (0.920-0.923)	0.197 (0.193-0.200)	1,809 (1806-1812)	15.1 (15.0-15.1)

Review	Seed	Total records	Relevant records	WSS@95% (CI)	n-WSS@95% (CI)	Precision@95% (CI)	Workload reduction in record numbers (CI)	Workload reduction in hours (CI)
	4	2000	0.025	0.860 (0.858-0.863)	0.927 (0.925-0.930)	0.210 (0.205-0.214)	1,821 (1816-1825)	15.2 (15.1-15.2)
	5	2000	0.025	0.841 (0.839-0.843)	0.907 (0.905-0.909)	0.174 (0.170-0.178)	1,782 (1777-1786)	14.8 (14.8-14.9)
Int2	1	500	0.1	0.577 (0.571-0.583)	0.663 (0.656-0.669)	0.203 (0.200-0.206)	314 (311-317)	2.6 (2.6-2.6)
	2	500	0.1	0.634 (0.631-0.638)	0.728 (0.724-0.732)	0.238 (0.236-0.240)	342 (340-344)	2.8 (2.8-2.9)
	3	500	0.1	0.672 (0.668-0.677)	0.771 (0.766-0.776)	0.277 (0.274-0.280)	361 (359-363)	3.0 (3.0-3.0)
	4	500	0.1	0.620 (0.616-0.625)	0.712 (0.707-0.717)	0.228 (0.226-0.230)	335 (333-337)	2.8 (2.8-2.8)
	5	500	0.1	0.603 (0.599-0.607)	0.692 (0.687-0.696)	0.219 (0.217-0.221)	326 (324-328)	2.7 (2.7-2.7)
Int2	1	1000	0.05	0.643 (0.637-0.649)	0.714 (0.708-0.720)	0.123 (0.120-0.126)	693 (687-699)	5.8 (5.7-5.8)
	2	1000	0.05	0.651 (0.648-0.655)	0.723 (0.719-0.726)	0.129 (0.128-0.130)	701 (698-705)	5.8 (5.8-5.9)
	3	1000	0.05	0.707 (0.700-0.713)	0.781 (0.774-0.788)	0.164 (0.160-0.168)	757 (750-763)	6.3 (6.3-6.4)
	4	1000	0.05	0.651 (0.647-0.655)	0.723 (0.719-0.727)	0.126 (0.125-0.128)	701 (697-705)	5.8 (5.8-5.9)
	5	1000	0.05	0.639 (0.635-0.643)	0.709 (0.705-0.714)	0.119 (0.117-0.120)	689 (685-693)	5.7 (5.7-5.8)
Int2	1	2000	0.025	0.685 (0.681-0.689)	0.746 (0.742-0.751)	0.072 (0.071-0.074)	1,470 (1461-1478)	12.2 (12.2-12.3)
	2	2000	0.025	0.672 (0.668-0.676)	0.733 (0.728-0.737)	0.069 (0.068-0.070)	1,444 (1435-1452)	12.0 (12.0-12.1)
	3	2000	0.025	0.715 (0.707-0.724)	0.778 (0.769-0.787)	0.087 (0.084-0.090)	1,531 (1513-1549)	12.8 (12.6-12.9)
	4	2000	0.025	0.653 (0.647-0.658)	0.713 (0.708-0.719)	0.064 (0.063-0.065)	1,406 (1395-1417)	11.7 (11.6-11.8)
	5	2000	0.025	0.666 (0.663-0.670)	0.727 (0.724-0.730)	0.066 (0.066-0.067)	1,433 (1427-1439)	11.9 (11.9-12.0)
Int4	1	500	0.1	0.710 (0.708-0.712)	0.813 (0.811-0.816)	0.313 (0.312-0.315)	380 (379-381)	3.2 (3.2-3.2)
	2	500	0.1	0.711 (0.708-0.713)	0.815 (0.812-0.817)	0.321 (0.319-0.323)	380 (379-382)	3.2 (3.2-3.2)
	3	500	0.1	0.700 (0.698-0.702)	0.802 (0.800-0.805)	0.302 (0.300-0.304)	375 (374-376)	3.1 (3.1-3.1)
	4	500	0.1	0.718 (0.716-0.720)	0.822 (0.820-0.825)	0.322 (0.321-0.324)	384 (383-385)	3.2 (3.2-3.2)
	5	500	0.1	0.705 (0.702-0.707)	0.808 (0.805-0.810)	0.298 (0.296-0.300)	377 (376-378)	3.1 (3.1-3.2)
Int4	1	1000	0.05	0.726 (0.723-0.729)	0.802 (0.799-0.805)	0.171 (0.169-0.173)	776 (773-779)	6.5 (6.4-6.5)
	2	1000	0.05	0.733 (0.731-0.735)	0.810 (0.807-0.812)	0.176 (0.175-0.177)	783 (781-785)	6.5 (6.5-6.5)
	3	1000	0.05	0.764 (0.762-0.766)	0.842 (0.840-0.844)	0.202 (0.200-0.204)	814 (812-816)	6.8 (6.8-6.8)
	4	1000	0.05	0.743 (0.741-0.745)	0.820 (0.818-0.822)	0.187 (0.186-0.188)	793 (791-795)	6.6 (6.6-6.6)
	5	1000	0.05	0.747 (0.745-0.748)	0.824 (0.822-0.826)	0.188 (0.187-0.189)	797 (795-798)	6.6 (6.6-6.7)
Int4	1	2000	0.025	0.738 (0.734-0.743)	0.801 (0.797-0.806)	0.092 (0.091-0.093)	1,577 (1568-1585)	13.1 (13.1-13.2)
	2	2000	0.025	0.764 (0.762-0.766)	0.828 (0.826-0.830)	0.104 (0.103-0.105)	1,629 (1625-1633)	13.6 (13.5-13.6)
	3	2000	0.025	0.764 (0.762-0.767)	0.828 (0.825-0.831)	0.103 (0.101-0.104)	1,628 (1623-1634)	13.6 (13.5-13.6)
	4	2000	0.025	0.757 (0.754-0.759)	0.820 (0.817-0.823)	0.100 (0.099-0.101)	1,613 (1608-1619)	13.4 (13.4-13.5)
	5	2000	0.025	0.774 (0.772-0.775)	0.838 (0.836-0.840)	0.108 (0.107-0.108)	1,648 (1645-1651)	13.7 (13.7-13.8)
Int6	1	500	0.1	0.539 (0.534-0.544)	0.619 (0.614-0.625)	0.187 (0.185-0.189)	295 (292-297)	2.5 (2.4-2.5)
	2	500	0.1	0.529 (0.520-0.537)	0.608 (0.598-0.617)	0.182 (0.179-0.186)	289 (285-294)	2.4 (2.4-2.4)
	3	500	0.1	0.581 (0.576-0.585)	0.667 (0.662-0.671)	0.200 (0.198-0.202)	315 (313-317)	2.6 (2.6-2.6)
	4	500	0.1	0.446 (0.438-0.453)	0.513 (0.504-0.522)	0.148 (0.145-0.150)	248 (244-252)	2.1 (2.0-2.1)
	5	500	0.1	0.586 (0.579-0.594)	0.673 (0.664-0.682)	0.213 (0.209-0.217)	318 (314-322)	2.6 (2.6-2.7)
Int6	1	1000	0.05	0.551 (0.544-0.558)	0.616 (0.609-0.623)	0.097 (0.095-0.099)	601 (594-608)	5.0 (4.9-5.1)
	2	1000	0.05	0.531 (0.522-0.540)	0.595 (0.585-0.604)	0.093 (0.091-0.095)	581 (572-590)	4.8 (4.8-4.9)
	3	1000	0.05	0.623 (0.617-0.630)	0.693 (0.686-0.700)	0.116 (0.113-0.118)	673 (667-680)	5.6 (5.6-5.7)
	4	1000	0.05	0.459 (0.450-0.469)	0.519 (0.508-0.529)	0.077 (0.075-0.079)	509 (500-519)	4.2 (4.2-4.3)
	5	1000	0.05	0.630 (0.625-0.635)	0.700 (0.694-0.705)	0.120 (0.118-0.121)	680 (675-685)	5.7 (5.6-5.7)
Int6	1	2000	0.025	0.567 (0.560-0.575)	0.625 (0.617-0.633)	0.051 (0.050-0.052)	1,235 (1219-1250)	10.3 (10.2-10.4)
	2	2000	0.025	0.524 (0.512-0.537)	0.581 (0.568-0.593)	0.046 (0.045-0.048)	1,149 (1125-1173)	9.6 (9.4-9.8)

Review	Seed	Total records	Relevant records	WSS@95% (CI)	n-WSS@95% (CI)	Precision@95% (CI)	Workload reduction in record numbers (CI)	Workload reduction in hours (CI)
	3	2000	0.025	0.649 (0.641-0.657)	0.709 (0.702-0.717)	0.064 (0.062-0.065)	1,398 (1383-1413)	11.7 (11.5-11.8)
	4	2000	0.025	0.477 (0.465-0.489)	0.532 (0.519-0.545)	0.041 (0.039-0.042)	1,054 (1029-1079)	8.8 (8.6-9.0)
	5	2000	0.025	0.620 (0.612-0.628)	0.680 (0.672-0.688)	0.059 (0.058-0.061)	1,340 (1325-1356)	11.2 (11.0-11.3)

Abbreviations: (n-)WSS: (normalized-)Work Saved over Sampling; CI: Confidence Interval.

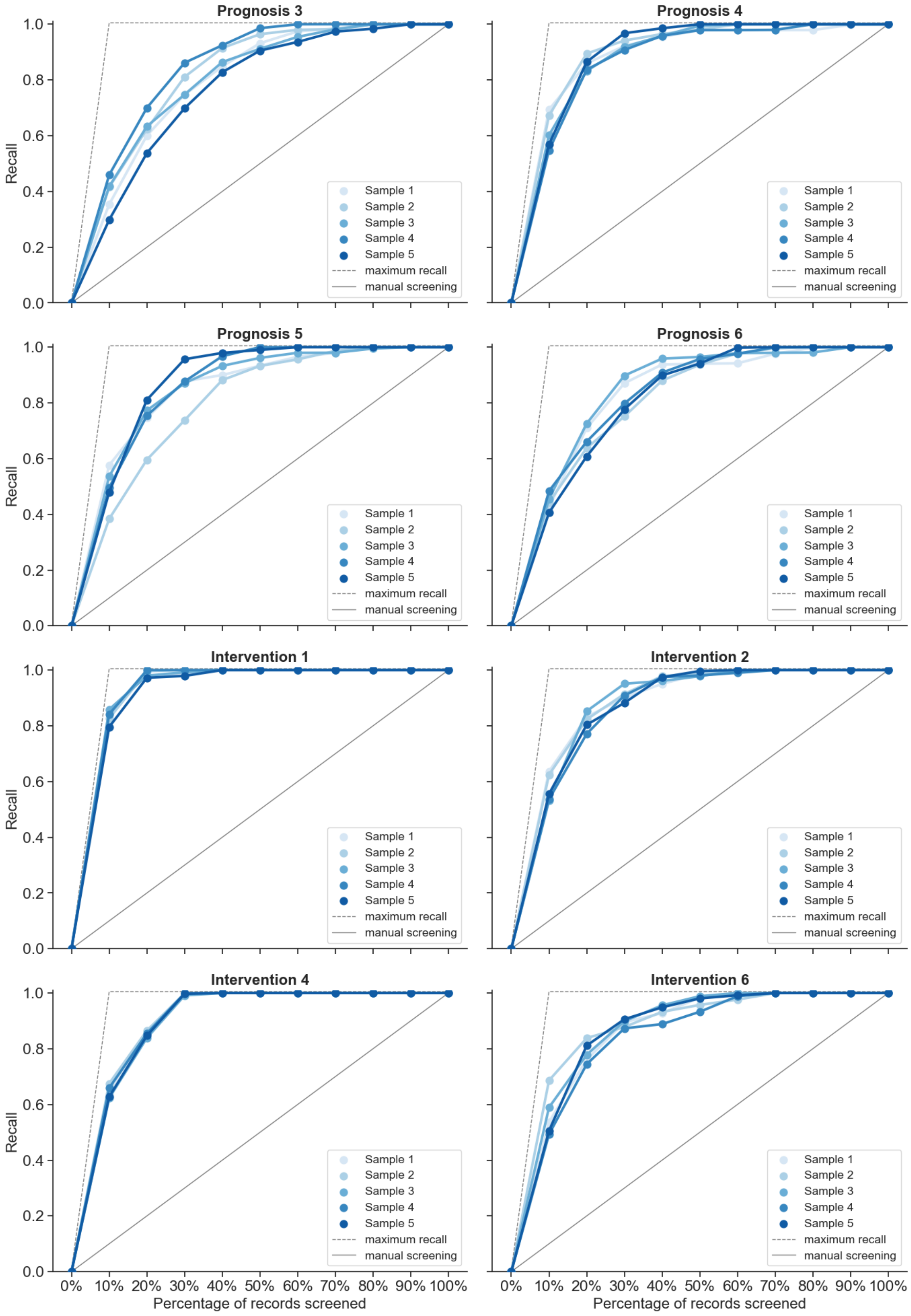

Figure A2 | Performance in terms of recall at increasing percentages of records screened with four of the prognosis and four of the intervention review datasets adapted to consist of 500 records of which 50 relevant (each sampled 5 times from the original datasets), and simulated with the default models (Term Frequency-Inverse Document Frequency (TF-IDF) + Naive Bayes (NB)) only

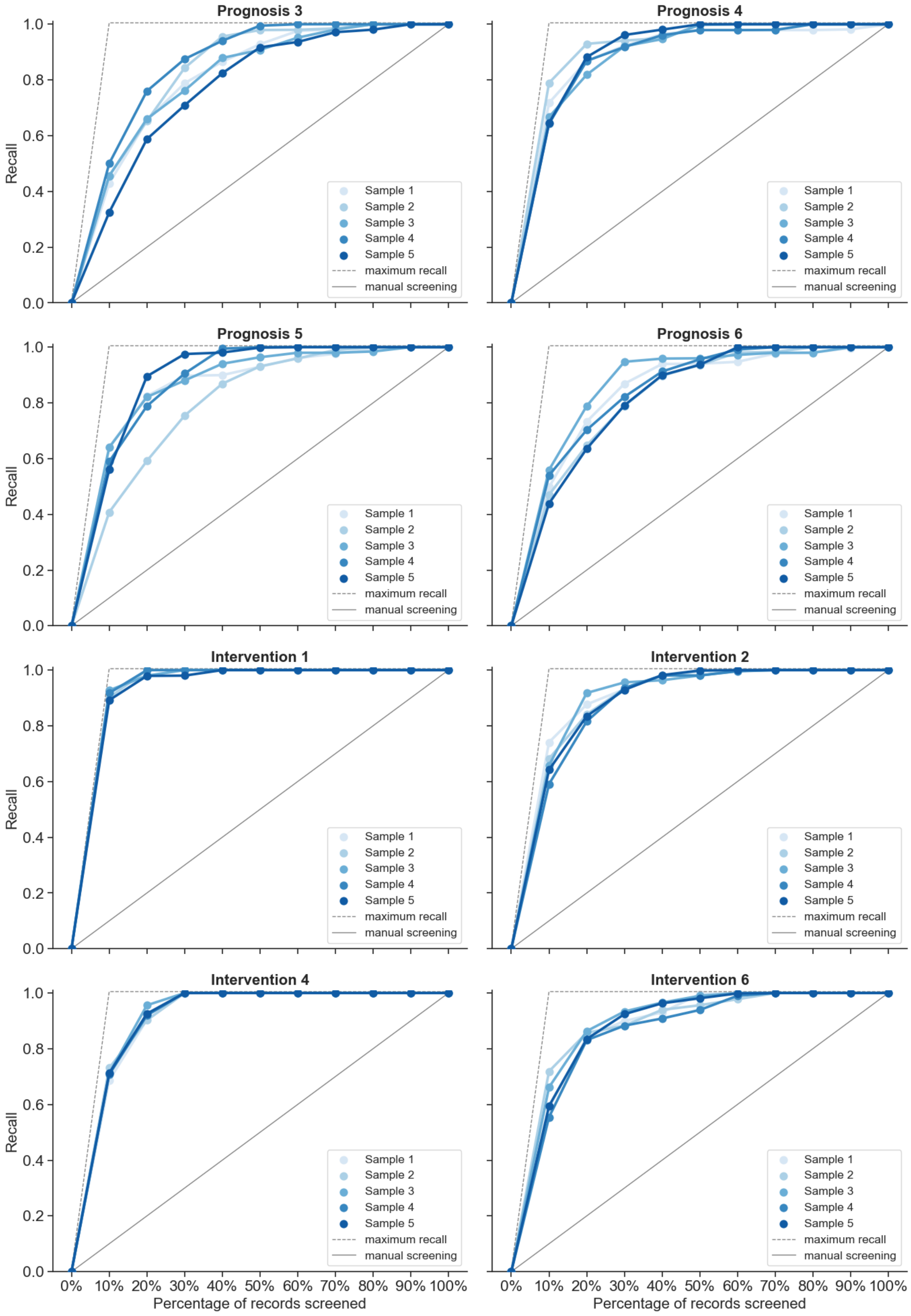

Figure A3 | Performance in terms of recall at increasing percentages of records screened with four of the prognosis and four of the intervention review datasets adapted to consist of 1000 records of which 50 relevant (each sampled 5 times from the original datasets), and simulated with the default models (Term Frequency-Inverse Document Frequency (TF-IDF) + Naive Bayes (NB)) only

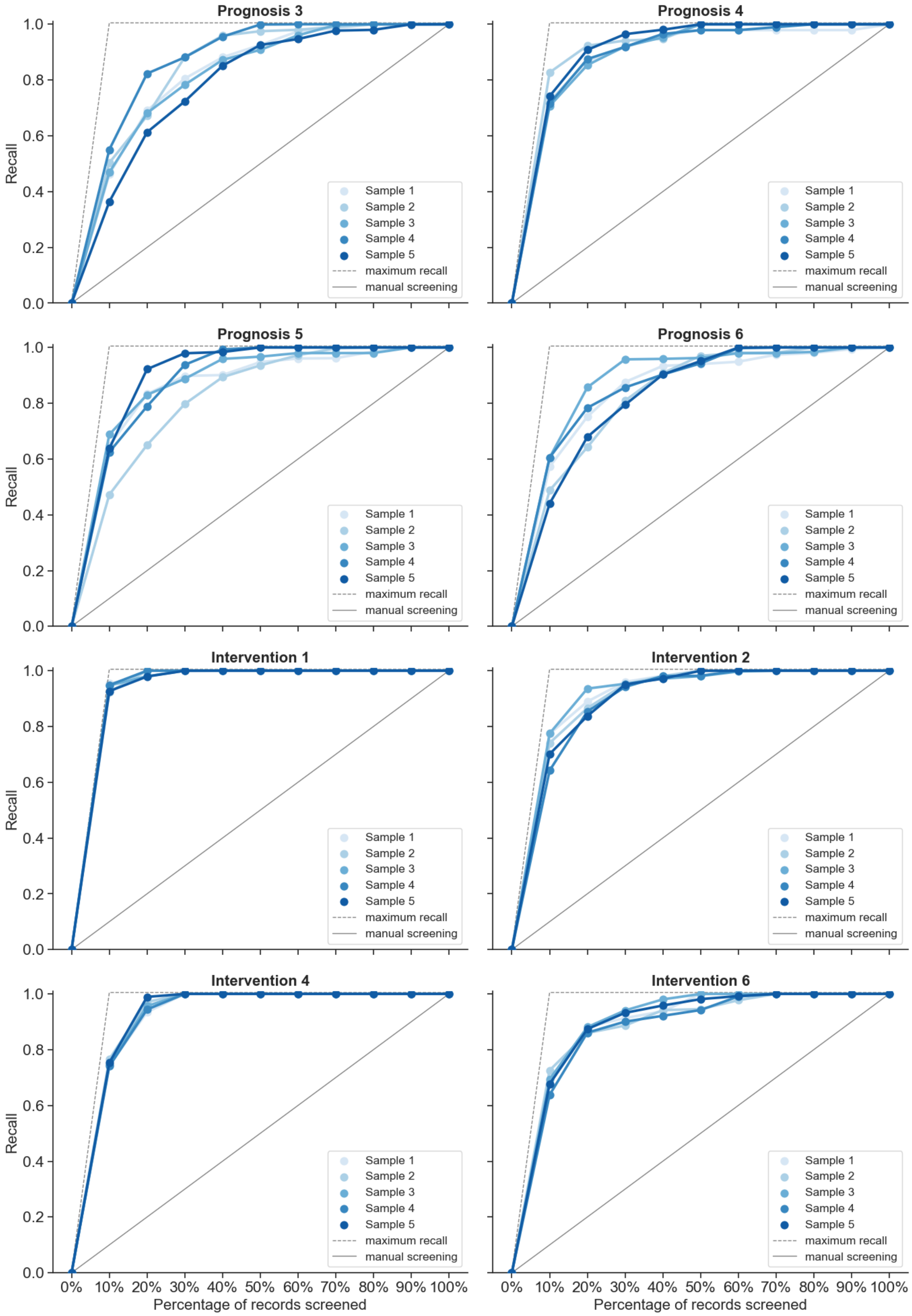

Figure A4 | Performance in terms of recall at increasing percentages of records screened with four of the prognosis and four of the intervention review datasets adapted to consist of 2000 records of which 50 relevant (each sampled 5 times from the original datasets), and simulated with the default models (Term Frequency-Inverse Document Frequency (TF-IDF) + Naive Bayes (NB)) only

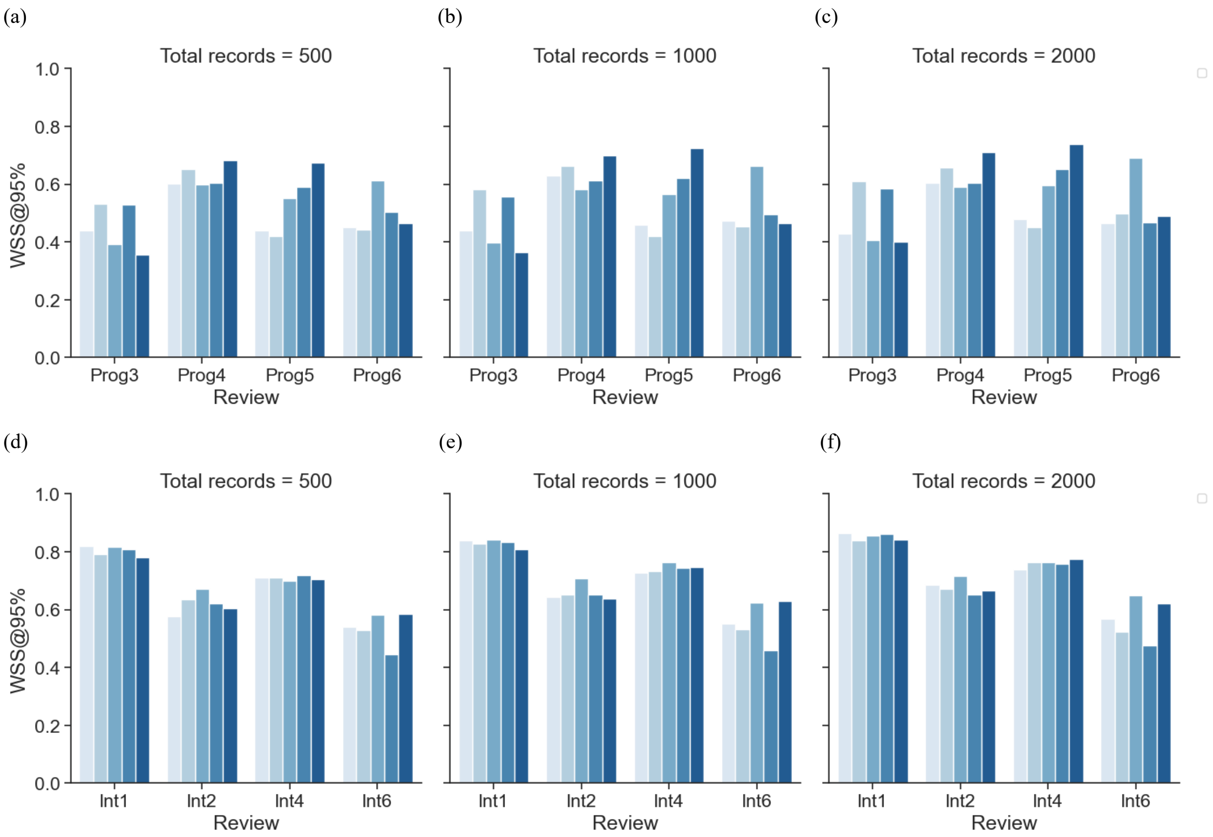

Figure A5 | WSS@95%recall for the simulations with default simulation models (Term Frequency-Inverse Document Frequency (TF-IDF) + Naive Bayes (NB)) and with manually adapted prognosis and intervention review datasets of 500, 1000, and 2000 records of which 50 relevant records, each sampled 5 times from the original datasets. (a-c) WSS@95% for the adapted prognosis reviews, (d-f) WSS@95% for the adapted intervention reviews. Abbreviations: WSS: Work Saved over Sampling; Int: Intervention; Prog: Prognosis

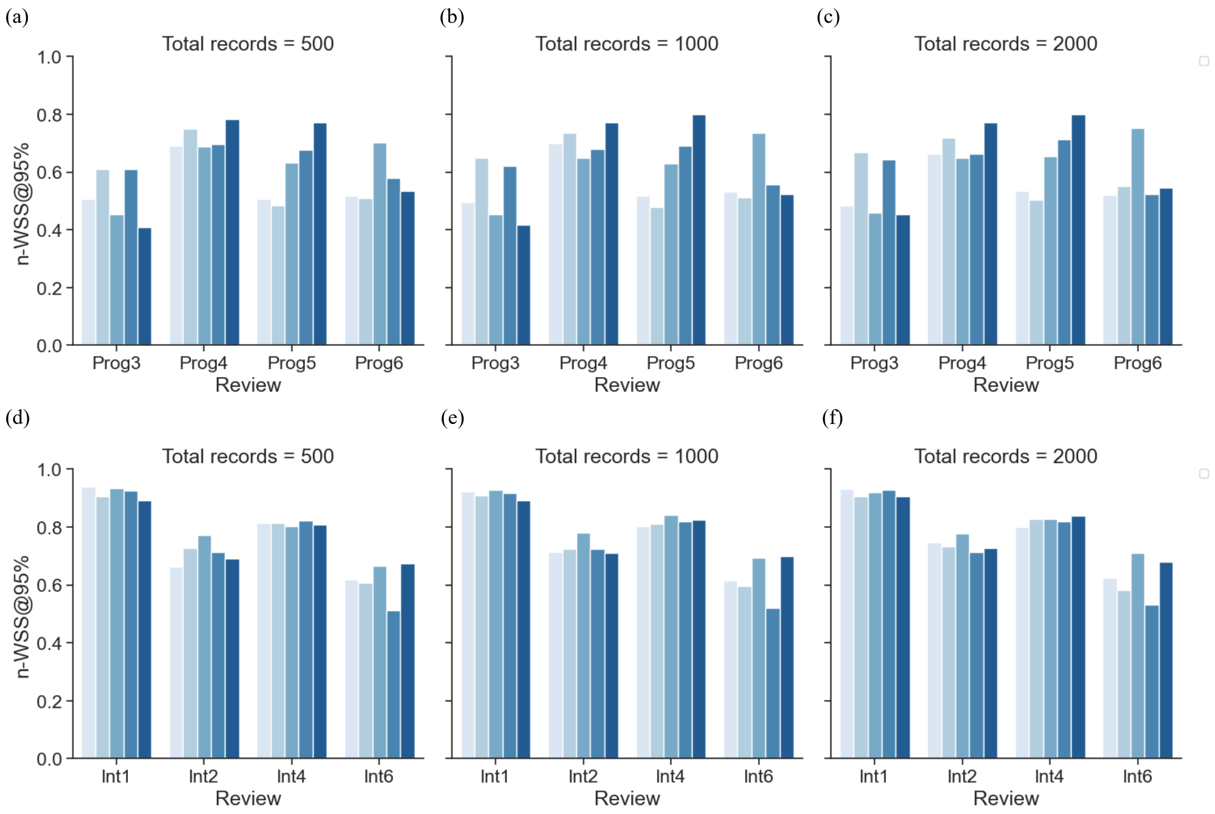

Figure A6 | n-WSS@95%recall for the simulations with default simulation models (Term Frequency-Inverse Document Frequency (TF-IDF) + Naive Bayes (NB)) and with manually adapted prognosis and intervention review datasets of 500, 1000, and 2000 records of which 50 relevant records, each sampled 5 times from the original datasets. (a-c) n-WSS@95% for the adapted prognosis reviews, (d-f) n-WSS@95% for the adapted intervention reviews. Abbreviations: n-WSS: normalized-Work Saved over Sampling; Int: Intervention; Prog: Prognosis

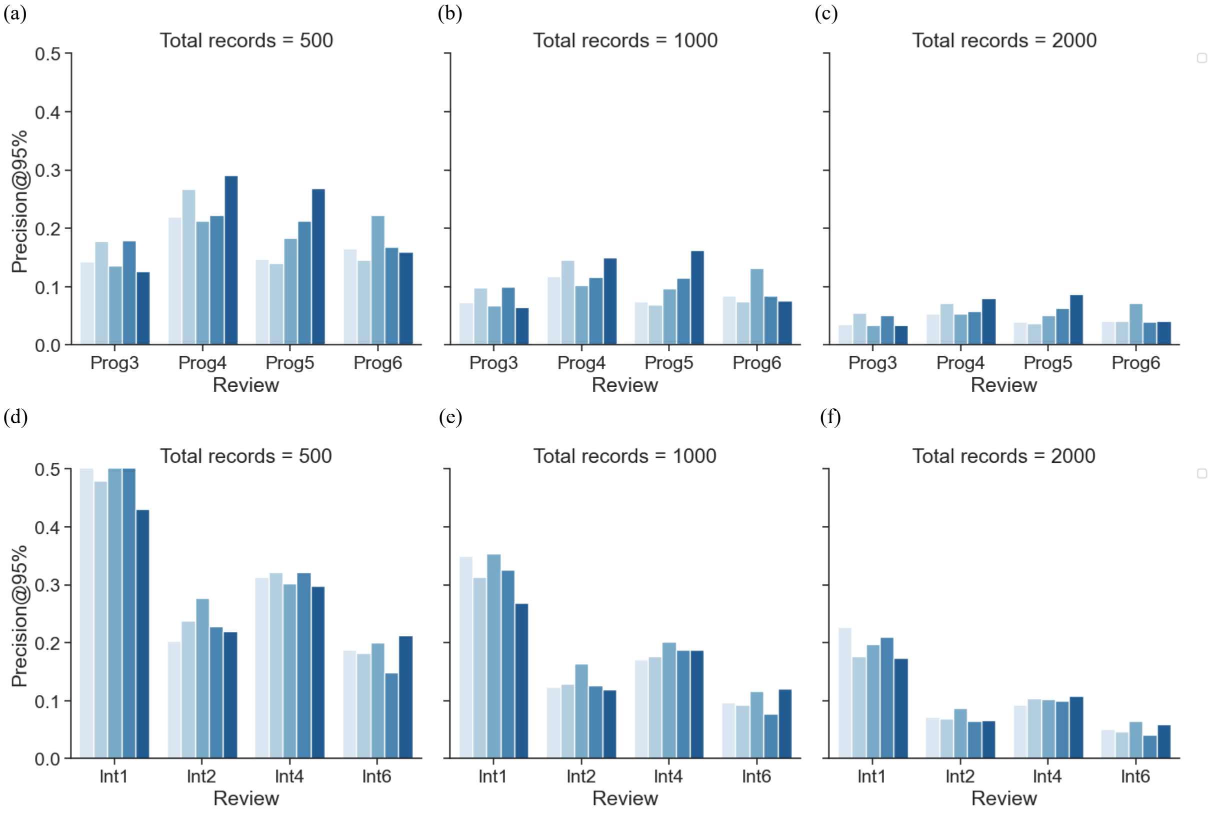

Figure A7 | Precision @95% recall for the simulations with default simulation models (Term Frequency-Inverse Document Frequency (TF-IDF) + Naive Bayes (NB)) and with manually adapted prognosis and intervention review datasets of 500, 1000, and 2000 records of which 50 relevant records, each sampled 5 times from the original datasets. (a-c) Precision@95% for the adapted prognosis reviews, (d-f) Precision@95% for the adapted intervention reviews.

Part IV: Results of intervention review A1 that did not meet the inclusion criteria

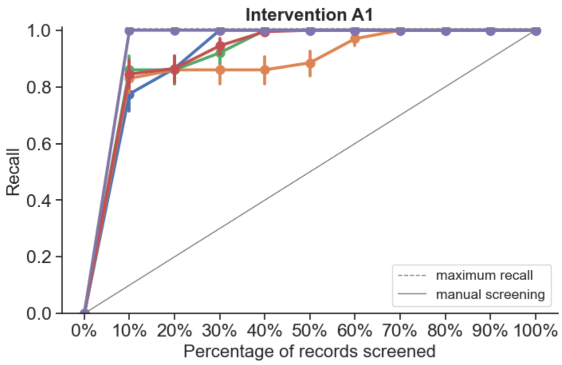

Figure A8 | Performance in terms of recall at increasing percentages of records screened for intervention review A1 that did not sufficiently meet the inclusion requirements of the study (i.e., this review had only 11 relevant records while the initial training of the models with default settings takes 10). Abbreviations: logistic: Logistic Regression; nb: Naive Bayes; SVM: Support Vector Machine; sbert: sentence Bidirectional Encoder Representations from Transformers; tfidf: Term Frequency-Inverse Document Frequency.

Table A3 | The raw and normalized Work Saved over Sampling at 95% recall (WSS@95% and n-WSS@95%) and the precision at 95% recall (precision@95%) for intervention review A1 with for each modelling method and averaged over 200 simulations

Review	Train model	Feature model	WSS@95% (CI)	n-WSS@95% (CI)	Precision@95% (CI)	Workload reduction in record numbers (CI)	Workload reduction in hours (CI)
IntA1	logistic	sbert	0.927 (0.924-0.930)	0.977 (0.974-0.980)	0.012 (0.010-0.014)	3,492 (3481-3502)	29.1 (29.0-29.2)
	logistic	tfidf	0.896 (0.882-0.911)	0.946 (0.932-0.961)	0.110 (0.095-0.125)	3,383 (3331-3434)	28.2 (27.8-28.6)
	nb	tfidf	0.902 (0.891-0.913)	0.952 (0.941-0.963)	0.105 (0.091-0.120)	3,402 (3362-3441)	28.4 (28.0-28.7)
	svm	sbert	0.885 (0.871-0.899)	0.935 (0.921-0.949)	0.008 (0.007-0.009)	3,341 (3291-3391)	27.8 (27.4-28.3)
	svm	tfidf	0.858 (0.831-0.884)	0.908 (0.881-0.934)	0.091 (0.077-0.105)	3,245 (3150-3339)	27.0 (26.3-27.8)

Abbreviations: (n-)WSS: (normalized-)Work Saved over Sampling; CI: Confidence Interval; logistic: Logistic Regression; nb: Naive Bayes; SVM: Support Vector Machine; sbert: sentence Bidirectional Encoder Representations from Transformers; tfidf: Term Frequency-Inverse Document Frequency.
